# Liposomal Encapsulation of Camptothecin/Carboxymethyl-β-Cyclodextrin Complexes: Stability, Solubility and Cytotoxicity

**DOI:** 10.3390/ijms27083705

**Published:** 2026-04-21

**Authors:** Fernando Mesías-Recamán, Alba Durán-Moreno, Thais Carmona, Gema Marcelo, Francisco Mendicuti

**Affiliations:** 1 Department of Chemical Engineering, University of Salamanca, Plaza Caidos S-N, 37008 Salamanca, Spain; fernando_bio_91@hotmail.com; 2 Institute of Biomedical Research of Salamanca (IBSAL), Paseo San Vicente 87, 37007 Salamanca, Spain; 3 Departamento de Química Analítica, Química Física e Ingeniería Química, Universidad de Alcalá, 28805 Alcalá de Henares, Spain; albitadm@hotmail.es (A.D.-M.); thais.carmona@uah.es (T.C.); 4 Instituto de Investigación Química “Andrés M. Del Rio” (IQAR), 28805 Alcalá de Henares, Spain

**Keywords:** cyclodextrins, fluorescence anisotropy, camptothecin, carboxymethyl-β-cyclodextrin, mono-(6-amine-6-deoxy) β-cyclodextrin, liposomes, molecular dynamics

## Abstract

The clinical utility of the anticancer drug camptothecin (CPT) is limited by its poor aqueous solubility and instability in the bloodstream, hindering bioavailability and efficacy. This study explores the complexation of CPT with carboxymethyl-beta-cyclodextrin (*cm*βCD) to overcome these limitations. Fluorescence spectroscopy and molecular modeling demonstrated 1:1 inclusion complexes, with stability constants governed by electrostatic interactions that were inversely correlated with pH. To validate this effect, a cationic amino-beta-cyclodextrin (*am*βCD) was used as a mechanistic control, revealing that Coulombic forces significantly modulate binding strength and stoichiometry. Crucially, *cm*βCD enhanced CPT solubility by up to 11-fold at 14 × 10^−3^ moldm^−3^, enabling a 385-fold increase in drug loading into liposomal carriers compared to the cyclodextrin-free system. Fluorescence-based release studies indicated high liposomal stability at physiological pH and partial CPT release under acidic conditions. Furthermore, CPT-loaded liposomes demonstrated cytotoxicity against cancer cell lines, particularly BT-474, with IC_50_ values generally comparable to or slightly higher than those of free CPT and the CPT:*cm*βCD complex, likely due to the distinct lysosomal cellular uptake pathway. This work highlights *cm*βCD complexation as a promising strategy to enhance CPT solubility and liposomal loading for improved drug delivery.

## 1. Introduction

**Scheme 1:**
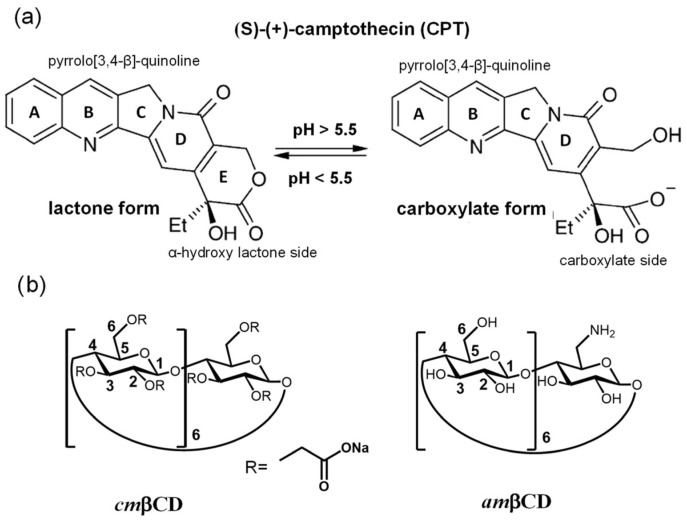
(**a**) Equilibrium between lactone and carboxylate forms of (S)-(+)-Camptothecin (CPT); (**b**) structures of carboxymethyl-β-cyclodextrin sodium salt (*cm*βCD) and mono-(6-amino-6-deoxy) β-cyclodextrin (*am*βCD) derivatives used in this work.

Camptothecin (CPT) is a fluorescent anticancer molecule belonging to the family of topoisomerase I inhibitors [1]. CPT has been mainly used for the treatment of stomach and bladder cancers [2]. CPT exhibits an intriguing structure–activity relationship, as it can exist in two distinct forms. The lactone form, which is stable at pH levels below 5.5, exhibits potent anticancer activity. However, at physiological (7.4) and alkaline pH, the lactone form readily hydrolyzes into the carboxylate form, resulting in a marked loss of its antitumor activity [2,3]. This conversion, which occurs in nearly 2 h in the bloodstream, results in a significant loss of antitumor efficacy [4]. The chemical structures of both forms of CPT are presented in Scheme 1.

CPT presents several important limitations for clinical application, primarily its low solubility in aqueous media [1] and the rapid lactone ring hydrolysis at physiological pH. In addition, the lactone form of CPT shows a high affinity for several components in the blood [5,6], particularly the human serum albumin (HSA), which reduces its bioavailability once introduced into the bloodstream [7,8]. In order to improve its solubility, synthetic analogues have been developed, of which two are used in chemotherapy, such as topotecan and irinotecan; however, their intrinsic activity is clearly lower than that of the pristine drug [9]. Consequently, enhancing CPT solubility while protecting its lactone ring without chemical modification remains a major challenge. In this context, cyclodextrins have emerged as effective systems [10].

Cyclodextrins (CDs) are cyclic oligosaccharides composed of α-D-glucopyranose units that form non-covalent inclusion complexes with organic molecules in their hydrophobic cavities. The incorporation of a wide diversity of covalently attached substituents on both the primary and secondary faces enable precise tuning of their inclusion properties. As a result, a wide range of CD derivatives can be synthesized and applied across different fields [11,12,13]. Particularly noteworthy are the applications of CDs in the pharmaceutical field, owing to their ability to efficiently encapsulate a wide variety of drugs [14,15,16].

Most of the studies have focused on the complexation of CPT with neutral CDs. Early investigations aimed at improving CPT solubility and stability were reported by Kang et al. [10] and Sætern et al. [17]. These studies demonstrated that native CDs and some neutral CD derivatives (e.g., HPβCD and randomly substituted dimethyl-β- and γCDs) form inclusion complexes with CPT, with stability constants ranging from 41 to 910 dm^3^ mol^−1^ and solubility enhancements between 5- and 170-fold. Both lactone and carboxylate isomers can form inclusion complexes; however, the ionized species typically exhibit lower affinity to the hydrophobic cavity of neutral CDs.

To address this limitation, the use of ionizable CDs is proposed to enhance solubility through complementary electrostatic interactions, a strategy that may also facilitate subsequent incorporation of CPT into nanocarriers at higher drug loadings.

To further improve pharmacokinetics and protect CPT from HSA binding, various nanocarriers for CPT have been explored [18,19,20,21,22,23,24,25,26,27,28], with liposomes being particularly advantageous due to their excellent biocompatibility [29,30,31]. Liposomes can encapsulate drugs of different physicochemical nature: those with a hydrophilic nature can be incorporated into the aqueous compartment of liposomes, while the most hydrophobic drugs can be incorporated into the lipid bilayers protecting them from degradation and improving their pharmacokinetics. As biocompatible and biodegradable systems, nearly twenty liposomal formulations have been approved to date for the clinical treatment of different diseases [30,31]. Drug-in-cyclodextrin-in-liposome (DCL) systems [32,33,34,35,36,37,38,39,40] have been developed to synergistically combine the solubility and stability enhancements in CDs with the biocompatibility and targeting capabilities of liposomes. This approach addresses the limitations of conventional nanoencapsulation by improving encapsulation efficiency, preventing burst release, and enabling the pH-controlled delivery of hydrophobic drugs.

Building on this rationale, the present work aims to enhance the aqueous solubility and stability of CPT without chemical modification. This is achieved by forming host–guest inclusion complexes with the pH-sensitive carboxymethyl-β-cyclodextrin (*cm*βCD) and subsequently incorporating the CPT:*cm*βCD complex into a liposome-based carrier. This dual-delivery strategy enables therapeutically relevant drug loading while protecting CPT. To accomplish this, we performed a comprehensive study focused on the following aspects:

i. Characterization of the complexation equilibrium of CPT with *cm*βCD: The thermodynamics and structural features of CPT complexation with *cm*βCD were investigated at pH 3.5 and 7.4 using fluorescence polarization and molecular modeling. To understand the role of electrostatic interactions, these results were compared with those obtained for the complexation of CPT with mono-(6-amino-6-deoxy)-β-cyclodextrin (*am*βCD).
ii. Effect of CPT complexation with *cm*βCD on drug solubility: The influence of CPT complexation with *cm*βCD on the drug’s solubility in aqueous solution was investigated at both pH values. In addition, stability constants were determined using phase solubility analysis.
iii. Development and characterization of stable liposomal systems with CPT: The experimental methodology focused on creating and characterizing stable liposomal systems containing CPT. This included investigating the encapsulation of free CPT and its complex with *cm*βCD in conventional liposomes using the passive loading method. Finally, we assessed the cytotoxicity of the different systems (CPT, *cm*βCD, CPT:*cm*βCD complex, and CPT complex loaded in liposomes) in vitro cell cultures of several cancer cell lines. Additionally, some CPT release/stabilization studies at both pH values were conducted.

## 2. Results and Discussion

### 2.1. Complexation of CPT with Carboxymethyl-β-Cyclodextrin (cmβCD)

#### 2.1.1. Absorption Spectra

The absorption spectra for CPT in PBS, citrate buffer and water solutions at pH 7.4 were obtained (Figure S1). In PBS and water, CPT showed a single absorption band centered at 370 nm. Conversely, in citrate buffer (pH = 3.5), CPT exhibited a double absorption band with maxima at 353 and 369 nm, along with a shoulder around 335 nm. The variation in spectral shape at different pH levels is consistent with previous studies of CPT under varying pH conditions [3,41,42,43]. The presence of increasing concentrations of *cm*βCD (approx. 0–12 × 10^−3^ moldm^−3^ range) did not significantly affect the position or intensity of the detected bands.

#### 2.1.2. Excitation and Emission Spectra

**Figure 1:**
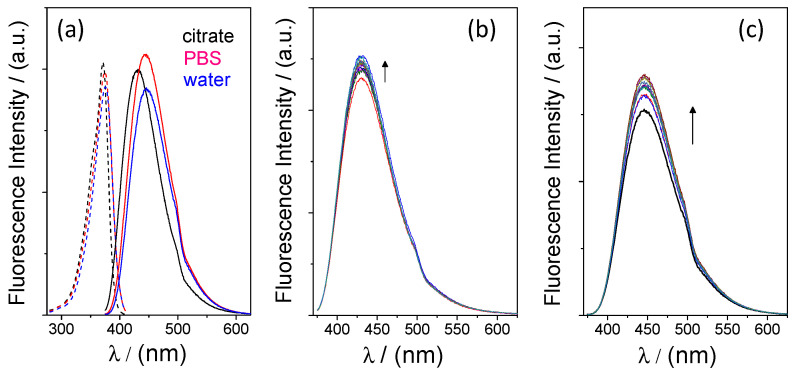
(**a**) Excitation (dashed lines) and emission (solid lines) spectra of CPT in absence of *cm*βCD in citrate buffer (pH = 3.5) (–), PBS saline (pH = 7.4) (–) and water (pH = 7.4) (–); (**b**) emission spectra of CPT in absence and in presence of increasing concentrations (from 0.42 to 11.97 × 10^−3^ moldm^−3^) of *cm*βCD in citrate buffer (pH = 3.5); (**c**) emission spectra of CPT in absence and in presence of increasing concentrations (from 0.42 to 12.08 × 10^−3^ moldm^−3^) of *cm*βCD in water (pH = 7.4). [CPT] was fixed at saturation 370 nm and the maxima of emission wavelengths in each solvent were used for monitoring the emission and excitation spectra, respectively. Measurements depicted were performed at 25.0 ± 0.1°C.

The emission spectra of CPT saturated solutions, both in the absence and presence of *cm*βCD in the three media studied depicted in Figure 1, showed a broad emission range from 380 to 625 nm. However, there were notable differences in the fluorescence emission spectra of CPT depending on the pH conditions. The emission maximum in citrate buffer with CPT in the lactone form was located around 431 nm without the appearance of additional peaks or shoulders. However, at pH of 7.4 (PBS or water), where the carboxylate form is present, the emission band presented an identical shape, but the maxima were red-shifted ~14 nm (located at ~445 nm) [3,42,44]. For increasing concentrations of *cm*βCD, the band positions in the emission spectra remain largely unaffected. However, a slight enhancement in intensity (as measured by the area under the emission spectrum) is observed for solutions in PBS or water at pH of 7.4. As illustrated in Figure S2a, this intensity increase is much less pronounced in citrate buffer. Similar trends were observed at other temperatures ranging from 5 °C to 45 °C in 10-degree intervals. Although the small changes in intensity with *cm*βCD concentration suggest the formation of a CPT-*cm*βCD complex, the uncertainties in measurements make it challenging to accurately quantify the association constants based solely on these intensity changes.

The fluorescence intensity decays for CPT/*cm*βCD solutions were obtained at the wavelength corresponding to the maximum of CPT emission bands (upon excitation at 370 nm). All intensity profiles were reasonably well-fitted to monoexponential decay functions. Figure S2b shows the changes in fluorescence lifetimes (τ) with increasing *cm*βCD concentrations, including the absence of *cm*βCD. While the fluorescence lifetime of CPT in aqueous solution and PBS buffer (carboxylate form) was approximately 4.2 ns, it decreased to nearly 3.7 ns in citrate buffer (lactone form). Importantly, varying concentrations of *cm*βCD had no significant impact on τ in any of these media [43].

In essence, the microenvironmental changes around CPT, potentially induced by complexation with *cm*βCD, have little to no significant effect on its fluorescence lifetime and only a slight effect on its intensity. To gain further insight into this behavior and substantiate this finding, fluorescence quantum yields (ϕ) and lifetimes (τ) were determined for dilute CPT solutions in media of varying polarity and viscosity. The obtained results (Section S3 and Figure S3) suggest that changes in the micropolarity and microviscosity of the medium encompassing CPT, whether in its free state or encapsulated within the *cm*βCD cavity, do not significantly alter its fluorescence lifetime or fluorescence intensity.

#### 2.1.3. Complexation of CPT with cmβCD

It is well-established that the complexation of a chromophore guest within a host molecule typically results in a reduction in its rotational diffusion coefficient, effectively increasing its rotational relaxation time. This phenomenon is often accompanied by a corresponding enhancement in fluorescence polarization (or anisotropy, *r*) [45]. Consequently, fluorescence anisotropy measurements can offer a precise and sensitive method for quantifying the binding of a guest molecule to a cyclodextrin host.

**Figure 2:**
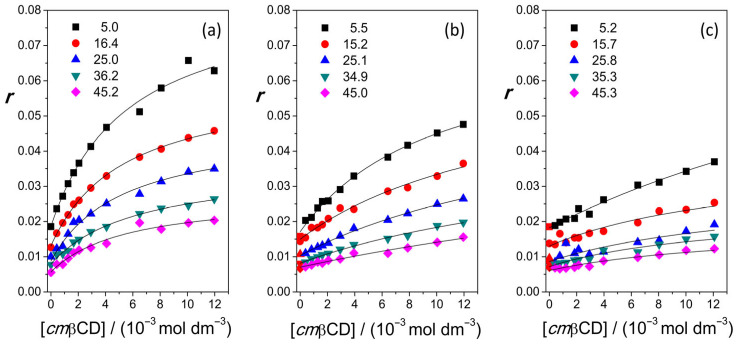
Variation in fluorescence anisotropy (*r*) for CPT solutions as a function of *cm*βCD concentration. Experiments were conducted in citrate (**a**), phosphate-buffered saline (PBS) buffers (**b**), and water (pH = 7.4) (**c**) at different temperatures. Measurements were performed at the maximum of emission upon excitation at 370 nm.

Figure 2 depicts the fluorescence anisotropy (*r*) in the absence and in the presence of increasing concentrations of *cm*βCD in citrate buffer (a), PBS buffer (b) and water (c). Upon increasing *cm*βCD concentration, the *r* values monotonically increased in all three media, suggesting the formation of inclusion complexes between CPT and *cm*βCD, which is correlated with a higher fraction of complexed CPT. The increase in temperature resulted in a decrease in *r*, probably due to a reduction in the fraction of CPT bound to *cm*βCD. Additionally, a minor contribution to this decrease could be attributed to an increase in the rotational diffusion rate of the entire CPT:*cm*βCD complex, likely caused by the lower viscosity of the medium. The observed increase in *r* for CPT bound to *cm*βCD is consistent with previous findings of restricted molecular motion for CPT bound to human serum albumin (HSA) and liposomes [5,6].

The experimental *r* data depicted in Figure 2 fits very well to Equation (S4) assuming a 1:1 stoichiometry. Linear adjustments to Equation (S5) also reasonably fit to a 1:1 stoichiometry. The association constants (*K*), which were relatively low, and *r_∞_* values (*r* at infinite host concentration, when all CPT is complexed) are collected in Table 1. In water at pH 7.4 and in PBS buffer, where the negatively charged *cm*βCD and CPT-carboxylate forms predominate, the values ranged from ~100 to ~20 dm^3^ mol^−1^, with no clear temperature-dependent trend due to the large uncertainties observed, particularly in water. These results suggest electrostatic repulsive interactions between charged *cm*βCD and CPT-carboxylate form diminished the association constants in PBS and water at physiological pH conditions. Previous studies have demonstrated a decrease in the stability constants between CPT and neutral HPβCD as pH increases, as determined by the phase solubility diagram [17]. However, in citrate buffer, the association constants showed higher values, ranging from 150 to 200 dm^3^ mol^−1^, without exhibiting a significant temperature dependence. These quantitative values are of the same order of magnitude as those previously obtained between non-charged CDs and the CPT-lactone form using the phase solubility method [10]. The *r_∞_* values (all guest complexed) were 0.047, 0.057 and 0.030 in citrate medium, PBS and H_2_O, respectively. If we assume the complex rotates as a single unit, the *r_∞_* value should be proportional to the size of the complex, which appears to be very similar across these media.

Thermodynamic parameters for the complexation of CPT and *cm*βCD were determined using classical linear van’t Hoff plots (see Figure S4). The results are summarized in Table 2. Significant differences were observed depending on whether the CPT-carboxylate or CPT-lactone form predominated in solution. These differences highlight how the inclusion process reflects a balance between hydrophobic interactions, electrostatics, and solvent reorganization.

In citrate buffer, where CPT remains predominantly in its lactone form, a Δ*H* ≈ 0 and positive entropy change (Δ*S* = +38.6 JK^−1^mol^−1^) were observed. This entropic profile is consistent with a hydrophobic inclusion mechanism: the complete insertion of the apolar lactone into the uncharged *cm*βCD cavity promotes the displacement of structured water molecules from both the inner cavity and the guest surface into the bulk solvent. Since this release of water molecules into a more disordered state outweighs the influence of specific host–guest interactions, the binding is strongly entropy-favored.

Under pH 7.4, where CPT exists mostly as the carboxylate form, the contribution of electrostatics becomes significant and the balance between enthalpy and entropy strongly depends on the medium. In PBS buffer, the complexation becomes enthalpy-driven (Δ*H* = −26.2 kJmol^−1^) with a large unfavorable entropy term (Δ*S* = –56.1 JK^−1^mol^−1^). The negative Δ*H* suggests the presence of favorable van der Waals and hydrogen-bonding interactions between CPT-carboxylate and the *cm*βCD cavity once electrostatic repulsion is efficiently screened by the high ionic strength. However, the entropic penalty likely reflects restricted conformational freedom and incomplete displacement of water molecules due to partial penetration of the charged guest into the cavity. In contrast, in water at pH 7.4, CPT binding becomes strongly entropy-driven (Δ*S* = +87.9 JK^−1^mol^−1^) but with an endothermic enthalpy contribution (Δ*H* = +16.2 kJmol^−1^). This unusual combination is compatible with significant electrostatic host–guest repulsion, leading to an enthalpic cost, which is overcompensated by a large entropic gain, which can be attributed to extensive desolvation of both species and the release of a highly ordered hydration shell surrounding the carboxylate group in the absence of ionic screening.

Quenching experiments from steady-state fluorescence measurements at 25 °C were also conducted in citrate buffer, PBS buffer and water, both with free CPT and CPT in the presence of *cm*βCD at the maximum concentration used in the previous experiments, using diacetyl (2,3-butanedione) as a quencher. The Stern–Volmer plots of fluorescence intensities derived from Equation (3) show an upward deviation from the linearity with increasing quencher diacetyl concentration (see Figure S5). The results obtained for the analysis of the first portion of the curve are summarized in Table S1. The bimolecular quenching rate constants (*k_q_*) obtained in the absence of the host, both in water and citrate buffer, were higher than those in PBS buffer. As expected, in all cases, the *k_q_* values were greater for free CPT compared to its complexed form. However, in water (pH 7.4) and citrate buffer, *k_q_* decreased by approximately 40% when comparing values in the absence and presence of *cm*βCD. In contrast, in PBS, the decrease was of around 20%. Consistent with the Δ*S* signs, these results suggest varying degrees of CPT inclusion within the *cm*βCD cavity. CPT appears to be deeply included in water and citrate buffer but not fully inserted in PBS buffer.

### 2.2. Complexation of CPT with Mono-(6-Amino-6-Deoxy) β-Cyclodextrin (amβCD)

Similar experiments were conducted in PBS and water at pH 7.4 to study the complexation of CPT with a positively charged βCD derivative, *am*βCD, which has an estimated pK_a_ of ~8.7 [46]. *am*βCD showed very poor solubility in citrate medium. Consistent with the previous *cm*βCD results, the emission spectra of CPT in water and PBS buffer displayed the same shape, with no substantial increase or decrease in intensity upon the addition of *am*βCD. Fluorescence lifetime decay profiles revealed mono-exponential behavior with approximately 4.3 ns lifetime values (τ) across all tested solutions, showing negligible dependence on *am*βCD concentration.

Similarly, the variations in *r* with [*am*βCD] at different temperatures in PBS buffer (a) and in water (b) were obtained (Figure S6). The *r* values also increased in the presence of *am*βCD and decreased with the temperature at each concentration. The sigmoidal shape with [*am*βCD] was indicative of a higher stoichiometry than 1:1. The association constants and *r_∞_*, values were obtained by fitting experimental anisotropy values to Equation (S4) by assuming 1:2 (CPT:*am*βCD) stoichiometries. The *K* values, summarized in Table S2, were 1000 times greater than those obtained for *cm*βCD in both media, consistent with the higher stoichiometry of the complexes. The *K*^1/2^ values were also larger than those observed for 1:1 CPT complexes with *cm*βCD. This can be attributed to attractive electrostatic interactions between the negatively charged CPT-carboxylate form and the positively charged *am*βCD, which favor higher stoichiometries and larger association constants [47]. Surprisingly, despite the larger complex size, the *r_∞_* values were similar to those obtained for the 1:1 CPT:*cm*βCD complexes. These relatively low values may be due to the fact that CPT, trapped between two *am*βCD host molecules, retains some degree of motion, meaning its movement is not entirely restricted.

The thermodynamic parameters obtained from linear van’t Hoff plots (see Figure S7) are summarized in Table 2. In both media studied, the complexation processes were both enthalpically and entropically favorable. The negative Δ*H* values indicate the presence of attractive interactions—primarily van der Waals contacts between the hydrophobic regions of CPT and the βCD cavities, together with favorable electrostatic interactions between the positively charged *am*βCD substituents and the carboxylate-form CPT framework. However, the observed magnitude of these enthalpic gains was smaller than typically expected, given the presence of favorable electrostatic interactions and the large complex stoichiometry, suggesting that part of the interaction energy is offset by structural penalties. In particular, the ternary complex formation where CPT is located between two *am*βCD units likely creates a relatively large and flexible supramolecular pocket. This enlarged cavity reduces the number and tightness of host–guest contacts, diminishing the overall van der Waals and electrostatic stabilization and explaining the small enthalpic contributions. In contrast, the substantial positive Δ*S* values point to a strong entropic driving force. This significant increase in entropy may be mainly due to the fact that CPT’s motion within the complex is only moderately restricted, as previously mentioned. This interpretation is further supported by the relatively low *r_∞_* values obtained.

### 2.3. Molecular Mechanics (MM) and Molecular Dynamics (MD) Simulations for CPT Complexation with cmβCD and amβCD

**Figure 3:**
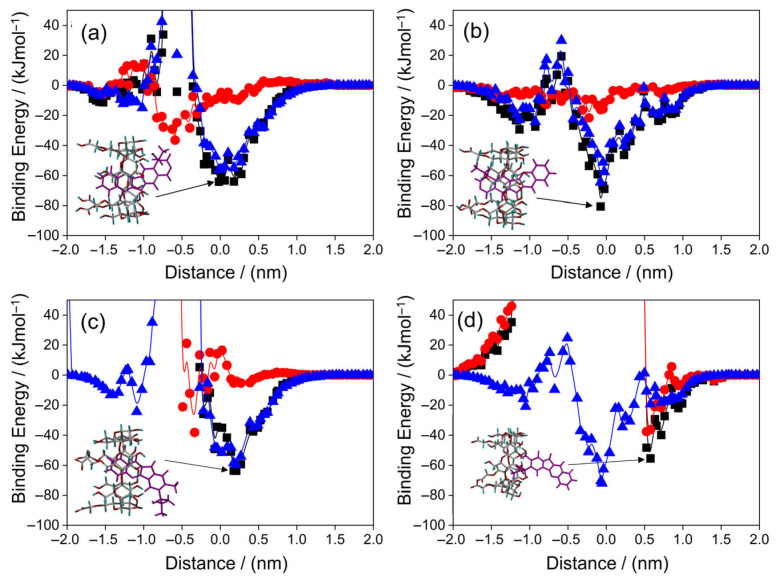
Variation in the total CPT–*cm*βCD interaction energies (■), electrostatic (●) and van der Waals (▲) contributions as CPT approaches the secondary face of uncharged *cm*βCD, either from the pyrrolo[3,4-β]-quinoline moiety (**a**) or the α-hydroxy lactone ring (**b**) sides of uncharged CPT. Idem for CPT approaching to the secondary face of charged *cm*βCD (−3 esu) from the pyrrolo[3,4-β]-quinoline (**c**) or the carboxylate (**d**) sides of charged CPT (−1 esu). Inset are the minima binding energy (MBE) structures indicated by the arrows.

Figure 3 illustrates the binding energy and its components as a function of the distance between the center of mass of CPT and *cm*βCD (scheme in Figure S21) mimicking the complexation of CPT with *cm*βCD at pH 3.5 and 7.4. As described in Section 3.9, the results represent two scenarios: (*i*) CPT in its lactone form (uncharged) approaching the secondary face of the uncharged *cm*βCD, either through the pyrrolo [3,4-β]-quinoline moiety (Figure 3a) or via the α-hydroxy lactone ring (Figure 3b) with a net system charge of zero, and (*ii*) CPT in its carboxylate form (−1 esu) interacting with the secondary face of the charged *cm*βCD (−3 esu), either through the pyrrolo[3,4-β]-quinoline moiety (Figure 3c) or via the open ring of the carboxylate form (Figure 3d), with a net system charge of −4 esu (for clarification see Scheme 1).

At pH 3.5, both CPT and cmβCD are neutral. The binding energy profiles during complexation, shown in Figure 3a,b, reveal a minimum binding energy (MBE) structure at an *oo’* distance close to zero. These structures, where a good portion of the guest penetrates inside the cavity, are reached without encountering any significant energy barrier, with the largest contribution to the total binding energy arising from van der Waals interactions, while electrostatic interactions contribute minimally. The most favorable MBE structure is achieved when CPT penetrates via the lactone side.

At pH 7.4, both CPT (−1 esu) and *cm*βCD (−3 esu) are negatively charged. The resulting electrostatic repulsion creates a barrier that hinders deep penetration of CPT. As expected, the most favorable and deepest inclusion occurs from the uncharged side, specifically via the pyrrolo[3,4-β]-quinoline moiety.

MD simulations of the four optimized MBE structures depicted in Figure 3, with the analysis shown in Figure S8, reveal that at pH 3.5, when both CPT and *cm*βCD are neutral, the structures formed by penetration through either the quinoline moiety (Figure S8a) or the lactone ring (Figure S8b) remain stable throughout the entire trajectory. These stable structures exhibit favorable binding energies, primarily driven by both electrostatic and van der Waals interactions, with a strong contribution from the latter. However, at pH 7.4, where both CPT and *cm*βCD are in their carboxylate forms, the complexes formed via penetration through either the quinoline moiety (Figure S8c) or the open ring of the carboxylate form (Figure S8d) remain stable only for the first quarter of the MD trajectory, after which the complex dissociates. As expected, electrostatic repulsion is primarily responsible for this instability.

Nevertheless, to further clarify the influence of electrostatic interactions on complex formation, the CPT with *am*βCD complexation was also investigated at both pH 3.5 and 7.4. The profile of binding energies and contributions as a function of the host–guest distances (Figure S22) during the approach is depicted in Figure S9. As expected, the favorable electrostatic contribution at pH 7.4 makes the complexation more energetically favorable (Figure S9c,d); however, complexation also seems possible at pH 3.5. In the latter case, van der Waals interactions are responsible for the stability of the complexes (Figure S9a,b). An analysis of MD simulations on the optimized MBE structures, which mimic the CPT:*am*βCD complexes obtained at pH 7.4 by either penetrating via the pyrrolo[3,4-β]-quinoline moiety (Figure S10c) or the carboxylate one (Figure S10d), whose complexation thermodynamics were experimentally studied, shows that both 1:1 complexes are stable throughout the trajectory. However, in both cases, a significant portion of CPT remained outside the CD cavity. This portion was subsequently forced to penetrate the secondary face of a second *am*βCD, forming a 1:2 stoichiometry complex as schematized in Figure S23 (see also Section 3.9). The changes in the total (CPT:*am*βCD)–*am*βCD and *am*βCD–*am*βCD interaction energies, as well as their contributions, were analyzed as the *am*βCD (+1 esu) approaches the preformed CPT:*am*βCD 1:1 complex, which has a net charge of zero (Figure S11). Both total interaction energies became slightly favorable as *am*βCD approached to a distance of approximately 0.5 nm, primarily due to van der Waals interaction. Beyond this point, a strong electrostatic repulsion, arising from CDs repulsion, occurred. The optimized 1:2 MBE stoichiometry structure (inset in Figure S11b) served as the starting structure for MD simulations. Analysis of the MD trajectory (Figure S12) revealed that the CPT:(*am*βCDs)_2_ complex remained stable throughout the simulation. Notably, while the pyrrolo[3,4-β]-quinoline moiety was located inside one of the *am*βCD, the carboxylate on the opposite side remained outside the second *am*βCD but interacted electrostatically with it. This interaction involved the protonated amino group at the primary face of the *am*βCD and the carboxylate moiety of CPT (Figure S13). This structural arrangement supports the experimentally obtained entropy changes.

### 2.4. Effect of CPT Complexation with cmβCD on Drug Solubility

This work emphasizes developing a vector for combining CPT with liposomes, aiming to provide new insights into how enhancing CPT solubility influences its loading into standard liposomal formulations. For this purpose, initially, in this section, we will present the results regarding the effect of solubilizing CPT in an organic solvent before mixing it with an aqueous solution containing *cm*βCD.

For this study, a fixed amount of the CPT was dissolved in MeOH and subsequently added to a *cm*βCD solution with different *cm*βCD concentrations (Section S14). It is noteworthy that the volume fraction of methanol in the final aqueous solution is notably low (~6%). This study was conducted at two pH values, 3.5 and 7.4, and different temperatures. The solubility phase diagrams provide an alternative way to obtain association constants of the inclusion complexes, as well as to determine the effect of increasing [*cm*βCD] on CPT solubility (see Section S15 for a description of the protocols). Notably, at 25°C, the 14 × 10^−3^ moldm^−3^ *cm*βCD solution at pH 3.5 enhanced CPT solubility by approximately 11-fold compared to its saturated aqueous solution at the same pH in the absence of *cm*βCD. Similar experiments were conducted at pH 7.4 (PBS); in this case, the solubility of CPT was increased by a factor of 6. However, it is important to note that the concentration of CPT in the saturated solution at pH 7.4 is higher than the concentration in the saturated solution at pH 3.5. Therefore, the concentration of CPT solubilized in the presence of *cm*βCD at pH 7.4 is larger than at pH 3.5.

A linear increase in CPT solubility was observed with *cm*βCD concentration at both pH values (Figure S15), with these curves being classified as of the A_L_ type of Higuchi and Connors [48], which indicate a CPT:*cm*βCD 1:1 stoichiometry. The stability constant of the complexes was calculated with Equation (S6) (Section S15). The binding constants were found to be 54 dm^3^ mol^−1^ and 208 dm^3^ mol^−1^ for pHs 7.4 and 3.5, respectively (see Figure S15 and Table S3), which are in good agreement with those determined by fluorescence anisotropy.

### 2.5. Development and Characterization of Stable Liposomal Systems with CPT

Conventional liposomes were prepared by combining phosphatidylcholine with cholesterol as described in Section 3.2. These systems were formed by hydrating a lipid film in PBS. After preparation, the liposomes were extruded through filters with 100 nm pore sizes to reduce size polydispersity. The final lipid concentration was 57.4 mg/mL. The liposomes exhibited a hydrodynamic diameter, expressed as a z-average, of 130 nm and a polydispersity index (PDI) of 0.06. These liposomes remained well dispersed with a zeta-potential of −21 mV and maintained their size for at least 21 days (Figure S16).

To encapsulate CPT, passive loading methods were employed, wherein liposomes were formed concomitantly with drug entrapment. For this purpose, the lipid film was hydrated using a saturated solution of CPT in PBS, resulting in a final lipid concentration of 57.4 mg/mL. Passive encapsulation techniques typically yield a substantial fraction of unencapsulated drug. Excess free CPT was eliminated by dialysis, and a standardized 30 h protocol was applied across all methodologies to ensure complete removal of non-encapsulated drug [49]. In this way, liposomes containing CPT with a molar concentration of 3.9 × 10^−8^ moldm^−3^ were obtained, and the encapsulation efficiency was 0.1%. Because the encapsulated concentration was quite low, this same methodology was employed with a solution of the preformed CPT:*cm*βCD inclusion complex in PBS at pH 7.4, in which the drug was dissolved with a concentration of 4.6 × 10^−5^ moldm^−3^ at a *cm*βCD concentration of 14 × 10^−3^ moldm^−3^. The presence of *cm*βCD permitted a notable increase in the solubility of CPT, as characterized above, in such a way that under these conditions, CPT was loaded in a significantly higher concentration of 1.5 × 10^−5^ moldm^−3^ (≈385-fold increase), and the encapsulation efficiency was increased up to 23.9%.

The release behavior of camptothecin from CPT:*cm*βCD-loaded liposomes was evaluated, as described in Section 3.6, by monitoring changes in fluorescence intensity under plasma-mimicking (pH 7.4) and lysosomal-mimicking (pH 3.5) conditions (Figure S17). At pH 7.4, fluorescence intensity remained essentially constant over time, indicating high stability of the liposomal formulation and negligible CPT leakage under physiological conditions.

At pH 3.5, a moderate and gradual increase in fluorescence intensity (*I*) of approximately 5–6% was observed, reaching a plateau after about 400 min. The linear relationship of ln (*I*_∞_ − *I*_t_) versus time suggests an apparent first-order process, with a rate constant k = 0.0103 min^−1^ and a corresponding half-life (τ_1_/_2_) of 67.3 min. This behavior is consistent with partial CPT release under acidic conditions.

Nevertheless, it should be emphasized that fluorescence intensity changes may be affected by several concurrent factors, including variations in the local microenvironment of the drug, pH-dependent changes in the photophysical properties and quantum yield of CPT, possible alterations in liposomal membrane permeability, and the coexistence of lactone and carboxylate forms of the molecule. Therefore, the fluorescence-based measurements should be regarded as qualitative and semi-quantitative indicators of CPT release rather than an absolute quantification. Despite these factors, the negligible fluorescence variation observed at pH 7.4 confirms the stability of the liposomal system in aqueous media at physiological pH.

In the following section, the cytotoxicity of free CPT, the CPT inclusion complex, and liposomes encapsulating the highest concentration of the CPT inclusion complex was evaluated. These assessments were performed using MTT assays across several cancer cell lines highly sensitive to CPT [50]: lung carcinoma (H-460), breast (BT-474), and colorectal carcinoma (HCT-116). The cytotoxic effect on healthy stroma cells (HS-5), was also investigated. Additionally, the cytotoxicity of *cm*βCD and lipid systems (without drug) was also determined in all cell lines.

We obtained the percentages of cell survival after exposing the different cell lines to *cm*βCD at a concentration of 1 mg/mL (0.65 × 10^−3^ moldm^−3^) (Figure S18). The percentage of cell survival remains close to 100% for all cell lines up to 48 h after exposure to *cm*βCD. This result shows that *cm*βCD does not possess cytotoxicity, which aligns with the literature. Unlike native β-cyclodextrin, which is cytotoxic, carboxymethyl- and hydroxypropyl-cyclodextrins exhibit minimal toxicity, making them suitable for pharmacological applications [51]. In addition, conventional liposomes also did not show cytotoxicity in the concentration range of this study (Figure S19).

**Figure 4:**
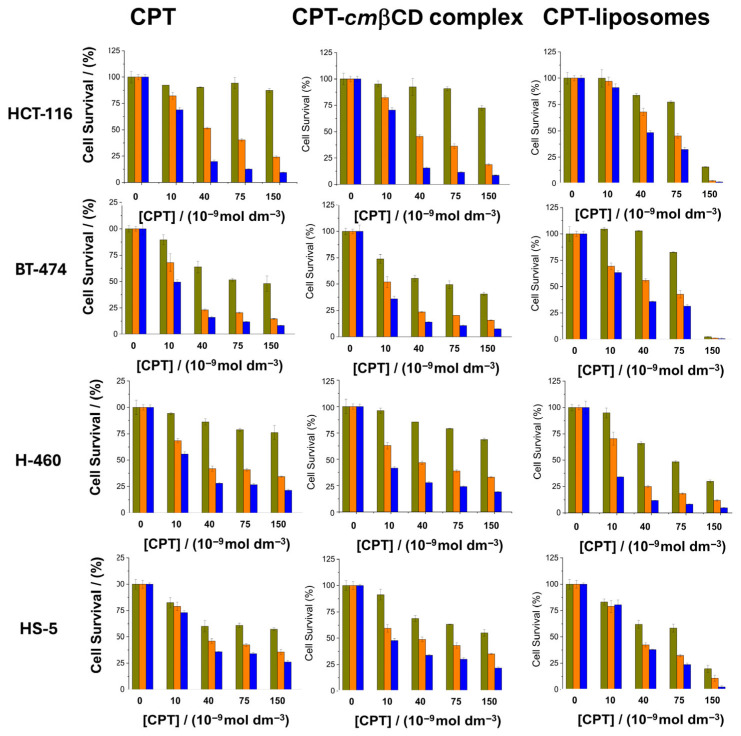
Cell survival percentage for the different cell lines up to 72 h after treatment with the different CPT systems depending on their concentration (■ 24 h, ■ 48 h and ■ 72 h).

Figure 4 shows the percentage of cell survival of the different cancer cell lines after treatment with free CPT and the CPT complex for different concentrations of CPT up to 150 × 10^−9^ moldm^−3^. In general, both camptothecin systems (free CPT and the CPT:*cm*βCD complex) exert a cytotoxic effect on all cell lines. At 24 h of treatment, the percentage of cell death exceeds 50% in the range of CPT concentration studied for the breast cancer line when the concentration of free CPT is greater than 75 × 10^−9^ moldm^−3^ and when the concentration of CPT in the presence of *cm*βCD is greater than 40 × 10^−9^ moldm^−3^. However, for a concentration of 40 × 10^−9^ moldm^−3^, the percentage of cell death exceeds 50% in all cell lines after 48 h of treatment. Half maximal inhibitory concentration (IC_50_) values were calculated after 48 h of treatment (Table 3).

The IC_50_ values for free CPT are within the range of 17–47 × 10^−9^ moldm^−3^, while those for the complex are slightly lower, falling within the 10–34 × 10^−9^ moldm^−3^ range. This observed increase in cytotoxicity cannot be attributed to *cm*βCD itself, as it showed no toxicity to the tested cell lines. However, increased cytotoxicity has been reported in the literature for some drugs administered with cyclodextrins, which has been attributed to the synergistic effect between cyclodextrin and the drug. In these cases, cyclodextrin would sequester cholesterol from the membranes, which would make cells more sensitive to the drug’s effect [52,53].

**Figure 5:**
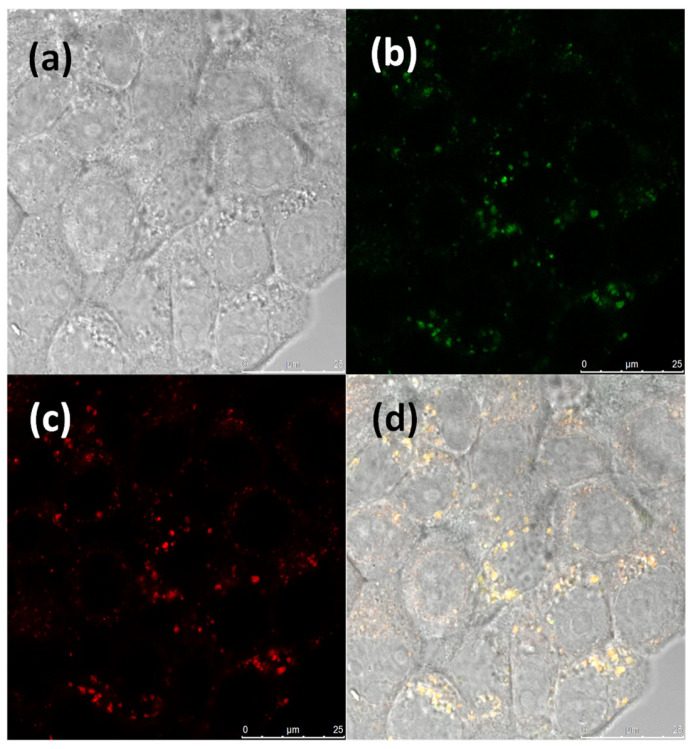
Fluorescence confocal images: (**a**) Bright field image. (**b**) Fluorescence image (excitation at 488 nm). (**c**) Fluorescence image (excitation at 570 nm). (**d**) Overlay images (**a**–**c**). Liposome concentration: 115 mg/mL.

Before assessing the cytotoxic effect of encapsulated CPT in liposomal systems, the entry mechanism of liposomes into breast cancer cells (BT474) was studied. To do this, the liposomes were labeled with a small percentage of fluorescent cholesterol (modified with the 25-NBD probe) and the lysosomes of the cells were stained with the Lysotracker red marker (see Section S20 for description of protocols). Fluorescence confocal images shown in Figure 5 (and Figure S20) (a: bright image of cells; b: fluorescence image with the fluorescence of the 25-NBD marked liposomes; c: fluorescence image with the lysosomes stained with Lysotracker; and d: overlay of images a, b and c) demonstrate that liposomes enter the cells, and that there is colocalization of their fluorescence with the fluorescence of the probe that stains the lysosomes. Therefore, this confirms that charged camptothecin enters lysosomes via an endocytosis mechanism.

Regarding the cytotoxicity of liposomes-CPT system, Figure 4 shows the percentage of cell survival of the different cell lines after treatment with liposomal formulations as a function of the concentrations of CPT administered. IC_50_ values for liposomes were found in a wide range: 18.6–67.8 × 10^−9^ moldm^−3^, the values for each cell line are collected in Table 3. As for CPT free or the CPT:*cm*βCD complex, the breast cancer line is the one on which there is a more important cytotoxic effect. For this line, the IC_50_ value was very similar to that obtained for the free drug (17.0 × 10^−9^ moldm^−3^).

The decrease in the cytotoxic effect of CPT when administered in liposomes could be due to the difference in the mechanism of entry into the cell; free CPT enters the cell by passive diffusion, a process that occurs at a higher rate than the process of endocytosis, the mechanism by which liposomes enter.

## 3. Materials and Methods

### 3.1. Materials

Commercially available (S)-(+)-campthotecin (CPT, Sigma-Aldrich, St. Louis, MO, USA) or Alfa Aesar (Ward Hill, MA, USA), purity ≥ 90%, CAS 7689-03-4) and carboxymethyl-β-cyclodextrin sodium salt (*cm*βCD, Sigma-Aldrich, water content of 4.9%, DS near 3, pK_a_ ≈ 6 [54], CAS 2828447-14-7) were used as received. The spectral purity of CPT was verified by the high degree of coincidence between UV-Vis absorption and fluorescence excitation spectra and further confirmed by the mono-exponential nature of its fluorescence intensity decays of CPT in different solvents, ensuring that minor impurities do not interfere with the photophysical measurements. Mono-(6-amino-6-deoxy)-β-cyclodextrin (*am*βCD, pK_a_ ≈ 8.7 [46], CAS 29390-67-8) was synthetized following the protocols reported by Tang et al. with minimal modifications [55] (Scheme 1). Milli-Q water (18.2 MΩcm) and organic solvents for fluorescence such as methanol (Sigma-Aldrich, ≥99.9%, CAS 67-56-1), ethanol (Uvasol^®^, spectrophotometric grade, ≥99.9%, CAS 64-17-5) and propanol (Sigma-Aldrich, ≥99.9%), CAS 71-23-8) were checked for impurities before use. pHs of 7.4 and 3.5 for the solutions were controlled using a phosphate-buffered saline solution (PBS) (NaCl 0.138 moldm^−3^; CAS 7647-14-5); KCl 0.0027 moldm^−3^; CAS 7447-40-7, Sigma-Aldrich or a citrate buffer (0.1 moldm^−3^ citric acid (77-92-9) and 1 moldm^−3^ NaOH (CAS 1310-73-2) in a 5:1 (*v*/*v*) ratio with HCl 0.1 moldm^−3^ (CAS 7647-01-0)), respectively. Additionally, milli-Q water pH was adjusted to 7.4 with NaOH and HCl solutions. Diacetyl (2,3-Butanedione, CAS 431-03-8) of 97% for quenching measurements was purchased from Sigma-Aldrich and used as received. Quinine hemisulfate salt monohydrate (Sigma-Aldrich, 99.0–101.0%, CAS 207671-44-1) in H_2_SO_4_ (Fluka, Seelze, Germany, aqueous solution 0.5 moldm^−3^, ≥99.9%, CAS 7664-93-9) was used as fluorescence standard (*ϕs* = 0.546) [56]. For solubility studies, methanol (MeOH, VWR Chemicals BDH (Radnor, PA, USA), >99.9%), acetic acid (AcH, 96% *v*/*v*, Scharlau (Barcelona, Spain, CAS 64-19-7) and dimethyl sulfoxide (DMSO, Sigma-Aldrich, ≥99.7%, CAS 67-68-5) were used.

Cells for the 3-(4,5-Dimethyl-2-thiazolyl)-2,5-diphenyl-*2H*-tetrazolium bromide (MTT, CAS 298-93-1) studies were supplied by American Type Culture Collection (ATCC, Manassas, VA, USA): H-460 (ATCC^®^ HTB-177^TM^): cells of lung carcinoma; BT-474 (ATCC^®^ HTB-20^TM^): cells isolated from a solid, invasive ductal carcinoma of the breast: HCT-116 (ATCC^®^ CCL-247^TM^): cells of carcinoma colorectal; and HS-5 (ATCC^®^ CRL-11882^TM^): stroma cells.

L-α-phosphatidylcholine (CAS 8002-43-5) and fluorescent cholesterol 25-[N-[(7-nitro-2-1,3-benzoxadiazol-4-yl)metil]amino]-27-norcolesterol (25-NBD, CAS 105539-27-3) was purchased from Avanti Polar Lipids Inc. (Alabaster, AL, USA), cholesterol (≥99%), dimethylsulfoxide (DMSO), Dulbecco’s Modified Eagle Medium (DMEM) and Red LysoTracker^TM^ were purchased from Merck, (Darmstadt, Germany), Petri dishes for confocal microscopy 35 mm height (Ibidi GmbH, Gräfelfing, Germany).

### 3.2. Lipid Film Formulations

The lipid film for liposome formation was prepared with 381 mg of phosphatidylcholine and 193.3 mg of cholesterol. The lipids were dissolved in 25 mL of chloroform (CAS 67-66-3), followed by solvent removal using a rotary evaporator for 2 h. The obtained lipid film was kept under vacuum for 5 h.

### 3.3. Solution Preparation for Film Lipid Hydration

Two CPT solutions were prepared: one was a saturated CPT solution in phosphate-buffered saline (PBS) at pH 7.4, and the other was a solution of CPT in PBS containing *cm*βCD. To prepare the saturated CPT solution, 3 mg of the compound was dispersed in 25 mL of PBS buffer at pH 7.4. The resulting suspension was continuously stirred for 12 h, and protected from light to minimize photodegradation of the active pharmaceutical ingredient. Subsequently, the solution was filtered through a 0.45 μm pore-size nylon membrane (Perkin Elmer, Shelton, CT, USA). The CPT concentration in the filtrate, quantified using ultraviolet-visible (UV-Vis) absorption spectroscopy, was 7.7 × 10^−6^ moldm^−3^. The CPT solution with *cm*βCD was prepared following a similar protocol: 1.7 mg of CPT was dissolved in 10 mL of a 14 × 10^−3^ moldm^−3^ *cm*βCD solution in PBS and stirred for 12 h under light-protected conditions, followed by filtration through a 0.45 μm nylon filter. The concentration of solubilized CPT in the resulting filtrate, determined by UV-Vis spectrophotometry, was one order of magnitude higher (4.6 × 10^−5^ moldm^−3^).

### 3.4. Methodology for the Formation of Liposomes Through Lipid Film Hydration with a CPT Solution

CPT solutions (with and without *cm*βCD) were used to hydrate the lipid film, in a manner that the CPT was encapsulated during liposome formation. The procedure consisted of: (*i*) hydration of the lipid film with 10 mL of CPT solutions; (*ii*) sonication of the resulting liposome suspension for 30 min to promote particle formation, followed by incubation in a water bath for two hours at 55 °C; after that, the liposome dispersion was extruded using an extruder (Avanti Polar Lipids Inc., Alabaster, AL, USA) equipped with polycarbonate filters with a pore size of 100 nm; and (*iii*) dialysis of the liposomes suspension against 200 mL of PBS for 30 h using a dialysis membrane (MWCO 3.5 kDa) was performed to remove non-encapsulated drug. During dialysis, the solvent was replaced once after the first 24 h. The final lipid concentration was 57.4 mgcm^−3^.

### 3.5. Determination of the Incorporated CPT Concentration

A 1 mL aliquot of the CPT-loaded liposome dispersion was initially treated with 3 mL of methanol to destabilize the lipid bilayer structure and release the encapsulated drug. This mixture underwent two consecutive centrifugation cycles at 7500 rpm for 14 min each, effectively separating lipid membrane debris from the unencapsulated CPT present in the supernatant. Then, the CPT content in the supernatant was analyzed by fluorimetry using calibration curves that correlated fluorescence intensity measurements with known CPT concentrations across a linear range. Encapsulation efficiency (EE) was determined by the ratio between the concentration of encapsulated CPT and the total initial CPT concentration.

### 3.6. CPT Release/Stabilization Studies

The release of camptothecin (CPT) from conventional liposomes was investigated using fluorescence spectroscopy. Release profiles were evaluated under plasma-mimicking and lysosomal-mimicking conditions at pH 7.4 and pH 3.5, respectively. Typically, 500 μL of the CPT-*cm*βCD-loaded liposomes ([CPT] = 3.46 × 10^−6^ moldm^−3^) prepared at pH 7.4 was dispersed into 2 mL of either phosphate-buffered saline (PBS, pH 7.4) or citrate buffer (pH 3.5). Fluorescence intensity was recorded immediately after dilution (time zero) and subsequently at different times until a constant signal was reached. All measurements were carried out at room temperature, and samples were protected from light throughout the experiments. Changes in fluorescence intensity were expressed as a percentage increase relative to the initial value.

### 3.7. Cellular Cytotoxicity Assays

Cells were seeded in 24-well plates at a rate of 6000 cells/mL for tumor cell lines and 25,000 cells/mL for healthy fibroblasts. They were incubated for 24 h for settlement and growth. The volume of each well was then removed and the same volume of culture medium containing the studied system (free CPT and loaded CPT in liposomes, or solution with CPT-cyclodextrin complex) was added. Each system was tested in triplicate. Subsequently, 100 μL of the MTT reagent was added to each well (final concentration of 0.45 mg/mL) and the plates were incubated for 1 h at 37 °C. The culture medium was then carefully removed, and 500 μL of DMSO was added to dissolve the formazan salts. The plates were placed on an orbital shaker for 10 min and protected from light to ensure homogenization of the resulting purple solution. The absorbance of each of the plates was measured in the spectrophotometer at a wavelength of 550 nm. Statistical analysis: each cell survival experiment consisted of three independent runs. For cell survival percentages, the arithmetic means and the error bars are presented in every bar graphic. A two-tailed Student’s *t*-test was utilized to analyze differences in IC_50_ values for each vector in the same cell line. The results were considered significant since they had *p*-values that were lower than 0.05.

### 3.8. Apparatus

Absorption spectra were monitored, using either a UV-Vis Uvikon 941 (Kontron Instruments, Milan, Italy) or a UV-1800 Shimadzu spectrophotometer (Shimadzu Corp., Kyoto, Japan). The temperature was controlled by a Huber MiniStat bath (Huber, Offenburg, Germany) or Varian Cary Peltier Temperature Controller (Varian Inc., Santa Clara, CA, USA), respectively. Steady-state fluorescence measurements were performed in a SLM Aminco 8100C spectrofluorimeter (SLM Instruments Inc., Urbana, IL, USA equipped with a double (single) monochromator in the excitation (emission) path. Slits were set at 8 nm and 4 nm for the excitation and emission paths, respectively. Except for polarization measurements, polarizers were fixed at the “magic angle” conditions. Detection was enabled by a photomultiplier cooled by a Peltier system. A PTI QuantaMaster time-correlated single-photon counting system (Photon Technology International, Birmingham, NJ, USA), adapted to incorporate monochromatic NanoLEDs (Horiba Jobin Yvon IBH Ltd., Glasgow, UK), was used for fluorescence decay measurements. An LED emitting at 370 nm served as the excitation source. Data acquisition was performed using the system’s integrated 1024-channel multichannel analyzer with a 100 ns time window. For each measurement, a total of 10,000 counts were collected in the maximum peak channel. The instrumental response function was regularly determined by measuring the scattering of a Ludox solution. Intensity fluorescence profiles were fitted to multi-exponential decay functions using an iterative deconvolution method [57].

The weighted average lifetime in case of a multiple-exponential decay function was defined as follows [58]:

(1) $$\langle \tau \rangle =\frac{\sum_{i=1}^{n}A_{i}\;\tau_{i}^{2}}{\sum_{i=1}^{n}A_{i}\;\tau_{i}}\;$$

where A_i_ is the pre-exponential factor of the component with a lifetime *τ_i_* of the multi-exponential decay function:

(2) $$I(t)=\sum_{i=1}^{n}A_{i}\;\tau_{i}$$

The fluorescence anisotropy, *r*, obtained from the fluorescence polarization measurements by using the *L-format* method, was defined as follows [45]:

(3) $$r=\frac{I_{VV}-GI_{VH}}{I_{VV}-2GI_{VH}}\;$$

where all magnitudes are well-known.

For a single isolated excited chromophore which is dynamically quenched by a quencher *Q*, the *I*_0_/*I* or τ_0_/τ ratios are related with [*Q*] by the linear Stern–Volmer equation:

(4) $$\frac{I_{0}}{I}=\frac{\tau_{0}}{\tau }=1+K_{SV\;}[Q]=1+k_{q}\;\tau_{0}\;[Q]$$

where *I*_0_ and *I* are the fluorescence emission intensities in the absence and presence of the quencher, respectively; *τ*_0_ and *τ* are the fluorescence lifetimes of the chromophore in the absence and presence of quencher Q; [Q] is the quencher concentration; *K_SV_* is the Stern–Volmer constant; and *k_q_* is the bimolecular quenching constant. The value of *k_q_* shows the accessibility of the quencher to the chromophore. For more complicated systems, the Stern–Volmer representations of <τ_0_>/<τ> are linear at the lowest [Q] region [59].

Particle size distribution and zeta potential were determined using a Zetasizer Nano series instrument (Malvern Panalytical Ltd., Malvern, UK). Formed liposomes underwent size reduction via sonication with a Starsonic 90 sonicator (Liarre Digit, Casalfiumanese, Italy). Cell internalization experiments were conducted using a fluorescence confocal microscope (Leica TCS-SP5, Leica Microsystems, Wetzlar, Germany), and the resulting images were analyzed with Leica LAS AF software (Version 2.7.3.9723, Leica Microsystems, Wetzlar, Germany).

Rectangular 10 mm path length cells (Hellma GmbH & Co. KG, Müllheim, Germany) or low-volume cylindrical cells with a 1.5 mm path length were used for all absorption and fluorescence measurements with *cm*βCD and *am*βCD solutions, respectively. In addition, polyethylene cuvettes were used to determine the size of the liposomes (Fisher Scientific, Thermo Fisher Scientific, Waltham, MA, USA) and zeta potential determination cuvettes (Zetasizer Nano series, Malvern, UK).

For spectroscopic measurements, solutions were prepared by weight using an analytical balance with a precision of ±0.1 mg. To ensure maximum accuracy in the concentrations, Class A volumetric glassware and calibrated micropipettes were used for all dilutions. The relative uncertainty in the concentrations of the prepared solutions was estimated to be lower than 2%. The temperature of the samples during fluorescence and UV-Vis measurements was controlled using the thermostatic systems described above with a stability of ±0.1 °C.

### 3.9. Theoretical Methods

Molecular mechanics (MM) and molecular dynamics (MD) calculations for the simulation of the complexation of CPT with *cm*βCD and *am*βCD were performed with Sybyl X2.0 [60] using the Tripos force field [61]. A relative permittivity ε = 1 was used in the presence of water and 3.5 in vacuo. The charges for the host molecules, *cm*βCD (SD = 3, net charge −3 esu or zero) and *am*βCD (SD = 1, net charge +1 esu), and for the guest molecule, CPT (net charge zero or −1 esu), were determined by MOPAC [62] and varied according to the simulated pH conditions. The starting modified βCDs were constructed with the macroring in a non-distorted conformation (ϕ = 0°, ψ = −3°, τ = 121.7°), and the rotational angles of the three methylcarboxylate moieties or the amine substituent at the primary face (position 6) were set in the *trans* conformation [63]. CPT, in both its lactone and carboxylate forms, was built atom-by-atom to ensure correct stereochemistry and connectivity. Optimizations were carried out by the simplex algorithm, and the conjugate gradient was used as the termination method with a gradient of 3.0 kcalmol^−1^Å^−1^ (0.2 kcalmol^−1^Å^−1^) in water (vacuo) [64,65]. Non-bonded cut-off distances were set at 12 Å. For water-solvated systems, the Molecular Silverware algorithm (MS) with periodic boundary conditions (PBCs) was used [66].

The basic protocol for the simulation of 1:1 complexes has been described previously [67]. Briefly, the CD derivatives, *cm*βCD or *am*βCD, were located with the center of mass of glycosidic oxygen atoms at the origin of the coordinate system and with the main axis oriented along the *y* axis (Figures S21 and S22). Next, a CPT molecule was approached to the secondary face of the CD along the *y*-axis in two different orientations: either through the pyrrolo[3,4-β]-quinoline moiety (ring A) or through the alpha-hydroxy lactone (ring E) or its hydrolyzed form, depending on the simulated pH conditions. At pH < 5.5, the alpha-hydroxy lactone form was used, and at pH > 5.5, the hydrolyzed lactone form was considered. The *cm*βCD derivative (pK_a_ ≈ 6) exhibited a net charge of −3 esu at pH 7.4 and was neutral at pH 3.5. In contrast, the *am*βCD derivative (pK_a_ ≈ 8.7 [46]), maintained a charge of +1 esu at both pH values.

Firstly, the most favorable CPT to CD relative orientation was previously optimized by obtaining binding energies for the structures generated by scanning the *O(4)-o-o*′*-O*′ dihedral angles, while the CPT distance was modified in small steps (Figures S21 and S22). These initials calculations were performed in the absence of explicit water (ε = 3.5 and gradient for optimization of each structure 0.2 kcal/molÅ). Fixed at the most favorable dihedral angle for any of the approaches, structures were generated by scanning *oo*′ distances from +20 to −20 Å at 0.5 Å intervals. Each structure was water-solvated (MS and PBC), optimized (gradient 3.0 kcal/molÅ) and saved for further analysis. The minimal binding energy (MBE) structure for each approach was optimized once again (gradient 0.5 kcalmol^−1^Å^−1^) and used as the starting conformation for the 1.0 ns MD simulations following the same strategy described earlier for similar systems [67,68,69,70,71,72,73,74].

The 1:2 stoichiometric CPT:(*am*βCD)_2_ complexes were generated by approaching an additional *am*βCD unit to the MBE 1:1 CPT:*am*βCD structure obtained in the previous calculations, as illustrated in the scheme of Figure S23. The remaining procedures followed were similar to those described for the 1:1 complexation.

## 4. Conclusions

The complexation equilibrium between camptothecin (CPT) and carboxymethyl-βCD (*cm*βCD) was investigated in citrate buffer (pH 3.5), phosphate-buffered saline (PBS, pH 7.4), and water (pH 7.4) using fluorescence spectroscopy, and the underlying processes were modeled via molecular mechanics (MM) and molecular dynamics (MD) simulations. Fluorescence anisotropy (*r*) titrations revealed the formation of 1:1 inclusion complexes between CPT and *cm*βCD in all three media. The stability of these complexes was found to be pH-dependent, correlating with the protonation state of CPT (lactone or carboxylate forms). Association constants (*K*) for the CPT-*cm*βCD complexes were significantly lower in PBS and water compared to citrate buffer, suggesting electrostatic repulsion between the negatively charged CPT carboxylate and the anionic carboxymethyl substituents of *cm*βCD. The crucial role of electrostatic interactions in modulating the association constant was further substantiated by a complexation study involving a protonated monoamine-βCD derivative (*am*βCD). At pH 7.4, this system exhibited a 1:2 stoichiometry CPT:*am*βCD complex, with K^1/2^ values greater than those observed for the 1:1 CPT-*cm*βCD complexes. This was attributed to favorable electrostatic attractions between the anionic CPT and the cationic *am*βCD. These experimental findings were supported by MM and MD calculations.

Furthermore, the presence of *cm*βCD significantly enhances the aqueous solubility of CPT. Notably, maximal CPT solubility was observed at a *cm*βCD concentration of 14 × 10^−3^ moldm^−3^, resulting in a 6-fold increase in CPT solubility under these conditions. Association constants obtained from phase solubility diagrams were in good agreement with those determined by fluorescence anisotropy.

The increase in solubility allows the complex to be protected in liposomes with sufficient CPT concentrations to have cytotoxic activity in different cancer cell lines as well as in healthy cells. The greatest cytotoxic effect was observed for the liposomes–CPT system on the breast cancer line (BT-474). The IC_50_ values for this system were in the 18.6–67.8 × 10^−9^ moldm^−3^ range and were higher than those determined for both free CPT and for the CPT:*cm*βCD complex. This is probably due to the different mechanism of entry of CPT into the cell (lysosome pathway) when it is protected by liposomes.

## Figures and Tables

**Table 1:** Association constants (*K*) and *r_∞_* values of the CPT and *cm*βCD at different media and temperatures. Obtained by fitting the experimental results to Equation (S4) in the Supplementary Materials (n fixed to 1).

Citrate (pH 3.5)			PBS (pH 7.4)			H_2_O (pH 7.4)		
T/°C	*r*_∞_	*K*/dm^3^ mol^−1^	T/°C	*r*_∞_	*K*/dm^3^ mol^−1^	T/°C	*r*_∞_	*K*/dm^3^ mol^−1^
5.0	0.086	176.8 ± 10.8	5.5	0.073	97.6 ± 15.7	5.2	0.098	25.5 ± 18.0
16.4	0.060	187.2 ± 3.7	15.2	0.064	59.5 ± 22.0	15.7	0.039	65.2 ± 52.1
25.0	0.047	190.5 ± 8.0	25.1	0.057	44.5 ± 10.1	25.8	0.030	61.2 ± 62.8
36.2	0.036	155.8 ± 6.7	34.9	0.044	40.3 ± 13.6	35.3	0.023	78.3 ± 53.6
45.2	0.028	177.4 ± 17.3	45.0	0.050	19.7 ± 22.3	45.3	0.019	66.9 ± 51.3

**Table 2:** Thermodynamic parameters obtained by van’t Hoff method using association constant values of Table 1.

System	Δ*H*/kJmol^−1^	Δ*S*/JK^−1^mol^−1^
CPT:*cm*βCD + citrate (pH = 3.5)	−1.3 ± 1.9	38.6 ± 6.6
CPT:*cm*βCD + PBS (pH = 7.4)	−26.2 ± 4.2	−56.1 ± 14.3
CPT:*cm*βCD + H_2_O (pH = 7.4)	16.2 ± 4.0	87.9 ± 25.2
CPT:*am*βCD + PBS (pH = 7.4)	−14.1 ± 4.0	40.2 ± 13.5
CPT:*am*βCD + H_2_O (pH = 7.4)	−3.0 ± 0.8	79.7 ± 2.9

**Table 3:** Half-maximal inhibitory concentration IC_50_ of the different systems with CPT: free CPT, CPT-*cm*βCD complex and conventional liposomes + *cm*βCD-CPT (CPT-liposomes) in the HCT-116 colon cancer, H-460 lung cancer, BT-474 breast cancer, and HS-5 healthy fibroblast cell lines.

	IC_50_ × 10^9^/moldm^−3^			
	HCT-116	H-460	BT-474	HS-5
CPT free	46.9 ± 4.4	24.5 ± 5.4	17.0 ± 2.9	40.9 ± 9.4
cm-CD-CPT complex	33.3 ± 6.1	24.3 ± 4.0	10.6 ± 1.6	22.6 ± 1.4
CPT-liposomes	67.8 ± 12.8	49.6 ± 3.1	18.6 ± 2.3	33.6 ± 2.9

## Data Availability

The original contributions presented in this study are included in the article/Supplementary Material. Further inquiries can be directed to the corresponding authors.

## Supplementary Materials

The following supporting information can be downloaded at: https://www.mdpi.com/article/10.3390/ijms27083705/s1. References [42,47,48,67,75,76,77,78,79] are cited in the Supplementary Material.

## Abbreviations

The following abbreviations are used in this manuscript:

25-NBD	25-[N-[(7-nitro-2-1,3-benzoxadiazol-4-yl)metil]amino]-27-norcolesterol
*am*βCD	mono-(6-amino-6-deoxy)-β-cyclodextrin
BT-474	cells of breast carcinoma
CD	cyclodextrin
*cm*βCD	carboxymethyl-β-cyclodextrin
CPT	camptothecin
DMSO	dimethyl sulfoxide
ϕ	fluorescence quantum yield
H-460	cells of lung carcinoma
HCT-116	cells of colorectal carcinoma
HPβCD	hydroxypropyl-βCD
HS-5	stroma cells
HSA	human serum albumin
IC_50_	half maximal inhibitory concentration
*K*	association constants
MBE	minimal binding energy (MBE)
MD	molecular dynamics
MM	molecular mechanics
MTT	3-(4,5-dimethylthiazol-2-yl)-2,5-diphenyltetrazolium bromide
MWCO	molecular weight cut-off
PBC	periodic boundary conditions
PBS	phosphate-buffered saline solution
PDI	polydispersity index
*r*	fluorescence anisotropy
UV-Vis	using ultraviolet-visible
τ	fluorescence lifetime
τ_1/2_	half-life for a first-order process
