# Supplementary material for: Liposomal Encapsulation of Camptothecin/Carboxymethyl-β-Cyclodextrin Complexes: Stability, Solubility and Cytotoxicity
Source: Int J Mol Sci. 2026 Apr 21;27(8):3705. doi: 10.3390/ijms27083705 (PMC13115757; doi:10.3390/ijms27083705)
Supplement: Supplementary file 1 [file ijms-27-03705-s001.zip › ijms-4235075-supplementary.pdf]

Liposomal Encapsulation of Camptothecin/Carboxymethyl-β-Cyclodextrin Complexes: Stability, Solubility and Cytotoxicity

**Table of Content**


Thermodynamics of the CPT-CD binding………………………………………… 5

Figure S1. Absorption spectra of CPT at the three different media: citrate buffer (pH=3.5) (–), PBS saline solution buffer (pH=7.4) (–) and water (pH=7.4) (–) at 25.0 ± 0.1 ºC…………………………………………………………………….. 6

Figure S2. (a) Normalized fluorescence ($I/I_0$) where I and $I_0$ are the areas under emission spectra measured at each [*cm*βCD] for CPT/*cm*βCD solutions and CPT in the absence of *cm*βCD respectively. (b) Lifetime ($\tau$) of CPT/*cm*βCD solutions in absence and in presence of *cm*βCD at different concentrations in citrate buffer (○), PBS buffer (□) and water (△). T=25±0.1 ºC…………………………….. 7

Section S3. Influence of the environment on the quantum yield and fluorescence lifetime of CPT……………………………………………………………….. 8

Figure S3. Fluorescence quantum yields (open symbols) and fluorescence lifetimes (full symbols) of CPT dilute solutions in methanol (MeOH), ethanol (EtOH), propanol (PrOH) and solvent mixtures: MeOH/$H_2O$ (50%), MeOH/$H_2O$ (90 %) and EtOH/$H_2O$ (58 %) (v/v) versus polarity (ε) (squares) and viscosity (η) (circles) of the solvents at 25 ± 0.1 ºC…………………………………………….. 9

Figure S4. Van't Hoff representations using association constants values of Table 1 for the complexation of CPT with *cm*βCD in tampon citrate at pH=3.5 (black squares), PBS and water at pH=7.4 (red circles and blue triangles respectively)………………………………………………………………… 10

Figure S5. Stern-Volmer plots from fluorescence intensities for isolated CPT (filled symbols) and at the maximum concentration used for *cm*βCD (~12 $\times10^{-3}$ moldm$^{-3}$) (open symbols) in citrate buffer (upper panel), PBS buffer (middle panel) and water (bottom panel)………………………………………………..... 11

Table S1. Stern–Volmer constants ($K_{SV}$), fluorescence lifetimes in the absence of diacetyl ($\tau_0$) and bimolecular quenching constants ($k_q$) for CPT in the absence and in the presence of *cm*βCD or *am*βCD in different solvents…………………… 12

Figure S6. Fluorescence anisotropies, *r*, for CPT/*am*βCD solutions in PBS buffer (a) and water at pH=7.4 (b) at near 5 (black), 15 (red), 25, (blue), 35 (green) and 45 (magenta) ºC. The concentration range were 0-12 $\times10^{-3}$ moldm$^{-3}$ in PBS and 0-6 $\times10^{-3}$ moldm$^{-3}$ in $H_2O$…………..……………………………………… 13

Table S2. Association constants ($K$) and $r_\infty$ values of the CPT and *am*βCD solutions in different media and temperatures obtained by fitting experimental values to Eq. S4 with n=2........................................................... 14

Figure S7. Vant'Hoff plots of Rln K vs $T^{-1}$ for the formation of CPT:*am*βCD complexes in PBS buffer (red circles) and water (blue triangles).................... 15

Figure S8. (a) Histories of the *oo´* distances between the center of mass of glycosidic oxygen atoms of *cm*βCD and the CPT (Figure S21) and binding energies (total binding in black, electrostatics in red and van der Waals in blue contributions), obtained from the analysis of MD simulations starting with the MBE structures of the CPT:*cm*βCD complexes obtained in MM (Figure 4 of the main text). Remember: for the approach of CPT to the secondary face of the uncharged *cm*βCD, either via the pyrrolo[3,4-β]-quinoline moiety (a) or the alpha-hydroxy lactone ring (b); idem for the approach of CPT to the secondary face of the charged (−3 esu) *cm*βCD, either via the pyrrolo[3,4-β]-quinoline moiety (c) or the open ring from the hydrolyzed lactone (carboxylate form) (d)... 16

Figure S9. Variation of the total CPT−*am*βCD interaction energies (■), electrostatic (●) and van der Waals (▲) contributions as uncharged CPT approaches the secondary face of charged *am*βCD (+1 esu), either from the pyrrolo[3,4-β]-quinoline moiety (a) or the α-hydroxy lactone ring sides of CPT (b) to mimic the complexation at pH 3.5. Idem for charged (−1 esu) CPT approaching to the secondary face of charged *am*βCD (+1 esu), from the pyrrolo[3,4-β]-quinoline (c) or the carboxylate (d) sides of charged CPT (−1 esu) mimicking the complexation at pH 7.4. Inset are the minima binding energy (MBE) structures indicated by the arrows.......................................... 17

Figure S10. Histories of the *oo´* distances between the center of mass of glycosidic oxygen atoms of *am*βCD and the CPT (Figure S22) and binding energies (total binding in black, electrostatics in red and van der Waals in blue contributions), obtained from the analysis of MD simulations starting with the MBE structures of the CPT:*am*βCD complexes obtained in MM ((Figure S9c and d) mimicking complexation at pH 7.4; Remember: for the approach of CPT to the secondary face of the protonated *am*βCD, either via the pyrrolo[3,4-β]-quinoline moiety (c) or the open ring from the hydrolyzed lactone (carboxylate form) (d).......................................................................... 18

Figure S11. (a) Variation of the total (CPT:*am*βCD)−*am*βCD interaction energies (■), along with electrostatic (●) and van der Waals (▲) contributions, as *am*βCD approaches the preformed CPT:*am*βCD 1:1 complex of charged *am*βCD (+1 esu) shown in Figure S10c following the approach scheme depicted in Figure S23. (b) Variation of the total *am*βCD−*am*βCD interaction energies (■), along with electrostatic (●) and van der Waals (▲) contributions during the formation of the 1:2 complex........................................................................ 19

Figure S12. Histories of several distances: (a) between center of mass of glycosidic oxygen atoms of both *am*βCDs (black); between nitrogen $-(NH_3)^+$ from nearest *am*βCD and carbon atom of the carbonyl group of the carboxylate

from CPT (red) and the *oo'* CPT to each *am*βCD1 (blue) or *am*βCD2 (green) distances; (b) Histories for the (*am*βCD)$_2$–CPT interaction energies (total in black, electrostatics in red and van der Waals in blue contributions), obtained from the analysis of MD simulations starting with the MBE structure of the CPT:(*am*βCD)$_2$ complex depicted in Figure S11b……………………………… 20

Figure S13. Structure for the CPT:(*am*βCD)$_2$ complex at the end of the molecular dynamic trajectory. By the arrow is shown the distance between nitrogen –$(NH_3)^+$ from nearest *am*βCD and carbon atom of the carbonyl group of the carboxylate moiety from CPT………………………………………………… 21

Section S14. Protocols for CPT solubility studies as a function of *cm*βCD concentration……………………………………………………………………... 22

Section S15. Phase-solubility diagrams protocols……………………………….. 22

Figure S15. Solubility diagrams of CPT in the presence of *cm*βCD at different temperatures and two pH values………………………………………………. 23

Table S3. Association constants (*K*) from solubility phase diagrams of Figure S15. Solubility of free CPT drug ($S_0$) at each pH and temperature were obtained in other experiments (standard deviation in *K* values of ~10%)………………………………………………………………………… 24

Figure S16. (a) Dynamic light scattering (DLS) measurements of liposomes dispersed in PBS buffer at 25 ± 0.1 °C. Three independent measurements were conducted over a 21-day period to monitor the evolution of the hydrodynamic diameter. The plot shown corresponds to one representative measurement of freshly prepared liposomes, illustrating the intensity-weighted hydrodynamic diameter distribution at 25 °C. The mean diameter was 133±40 nm (intensity-weighted average).(b) Time-dependent variation of the mean hydrodynamic diameter of liposomes stored in PBS at 25 °C over 21 days……………………… 25

Figure S17. Time-dependent fluorescence intensity increase (%) representing CPT release from liposomes at pH 3.5 (●) and pH 7.4 (○). The system exhibits pH-responsive behavior with significant release at acidic conditions. Inset: Linearized first-order kinetic plot for pH 3.5, showing $\ln(I_{inf}- I_t)$ vs. time (min). The linear correlation confirms that the release of CPT follows first-order kinetics ($k=0.0103$ $min^{-1}$, $\tau_{1/2}=67.3$ min)……………………………………………….. 26

Figure S18. Cellular viability after exposing the different cell lines to *cm*βCD at a concentration of 1mg/mL ($0.65 \times 10^{-3}$ $moldm^{-3}$)…………………..………… 27

Figure S19. Cellular viability after exposing the different cell lines to different liposome concentration necessary to attain the CPT concentration of X scale………………………………………………………………………… 28

Section S20. Protocols for Cell Lysosome Staining…………………………... 29

Figure S20. Fluorescence confocal images: a) Bright field image. b) Fluorescence image (excitation at 488 nm). c) Fluorescence image (excitation at 570 nm). d) Overlay images a, b and c. Liposomes concentration 115 mg/mL……………… 30

Figure S21. (A) Scheme for the approach of CPT to the secondary face of the uncharged *cm*βCD, either via the pyrrolo[3,4-β]-quinoline moiety or the alpha-hydroxy lactone ring, simulating conditions at pH 3.5 (net system charge of 0 esu). (B) Similarly, for the approach of CPT to the secondary face of the charged (−3 esu) *cm*βCD, either via the pyrrolo[3,4-β]-quinoline moiety or the open ring from the hydrolyzed lactone (−1 esu), simulating conditions at pH 7.4 (net system charge of −4 esu)………………………………………………………………………… 31

Figure S22. (A) Scheme for the approach of CPT to the secondary face of the charged (+1 esu) *am*βCD, either via the pyrrolo[3,4-β]-quinoline moiety or the alpha-hydroxy lactone ring, simulating the conditions at pH 3.5 (net system charge of +1 esu). (B) Similarly, for the approach of CPT to the secondary face of the charged (+1 esu) *am*βCD, either via the pyrrolo[3,4-β]-quinoline moiety or the open ring from the hydrolyzed lactone (−1 esu), simulating the conditions at pH 7.4 (net system charge of 0 esu)……………………………………………………………. 32

Figure S23. Scheme for the approach of a second amβCD molecule to the CPT βCD MBE structure of the (1:1) complex via the pyrrolo[3,4-β]-quinoline moiety, simulating the formation of a 1:2 stoichiometric complex at pH 7.4 (with a net system charge of +1 esu)…………………………………………………………… 33

## Thermodynamics of the CPT-CD binding

For a $CD_n$:G complex, with stoichiometry 1:*n*, whose global equilibrium can be written as:

$$nCD + G \rightleftharpoons CD_n : G \tag{S1}$$

where *K* is the association constant:

$$K = \frac{[G:CD]}{[G][CD]^n} \tag{S2}$$

where G is the guest molecule (CMP). Under the assumption that $[CD]_0 \approx [CD]$ and a single complex $CD_n$:G exists (or that) $[G:CD_n] \gg [G:CD_{n-1}] \gg \ldots \gg [G:CD]$, the molar fraction of complexed G, $x_2$ ($\neq f_2$) can be written as

$$x_2 = \frac{[CD_n:G]}{[G]_0} = \frac{K\,[CD]_0^n}{1 + K\,[CD]_0^n} \tag{S3}$$

where *K* is the association or complex formation constant for the equilibrium.

The molar fraction $x_2$ as a function of [CD] can be obtained from the change in a spectroscopic fluorescence derived property *Y* (emission fluorescence as the area under emission spectrum, I, average lifetime, <τ> or anisotropy for example) upon complexation. *Y* may significantly change from $Y_0$ (free guest) to $Y_\infty$ (complexed guest). By relating $x_2$ to *Y*, the reorganization of Equation (S2) might give us the following general expression,

$$Y = \frac{Y_0 + Y_\infty \Phi K [CD]_0^n}{1 + \Phi K [CD]_0^n} \tag{S4}$$

where Φ, which has different meanings depending on *Y*, is related to the change in fluorescence quantum yield and molar absorptivities for the free and complexed forms and Φ=1 when the property measured is the fluorescence intensity or the anisotropy. For average lifetime, <τ> then $\Phi = \frac{I_\infty}{I_0}$ (measured at the excitation and emission intensity of the lifetime measured decay profiles) [1].

Equation S4 can be expressed in linear form,

$$\frac{[CD]_0^n}{(Y - Y_0)} = \frac{1}{(Y_\infty - Y_0)K} + \frac{[CD]_0^n}{(Y_\infty - Y_0)} \tag{S5}$$

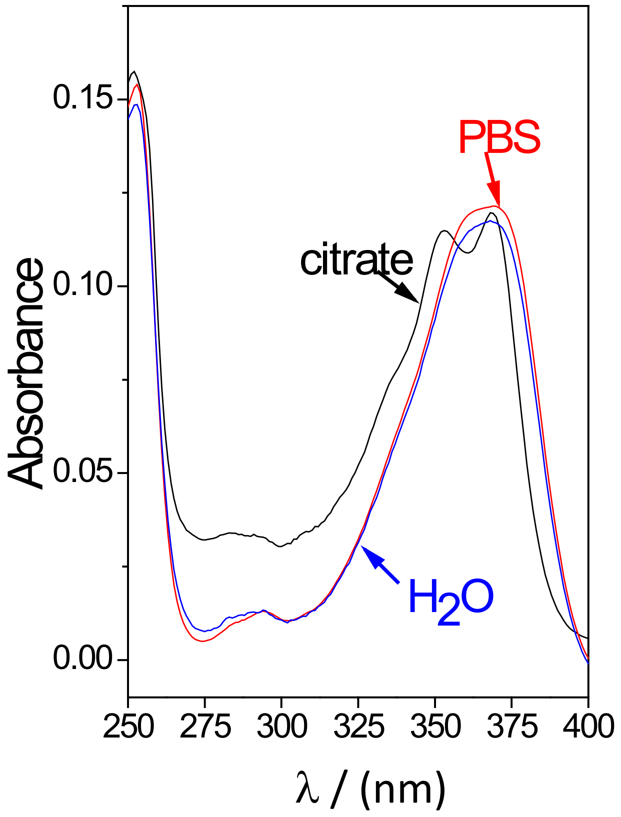


Figure S1. Absorption spectra of CPT at the three different media: citrate buffer (pH=3.5) (–), PBS saline solution buffer (pH=7.4) (–) and water (pH=7.4) (–) at 25 ± 0.1 ºC.

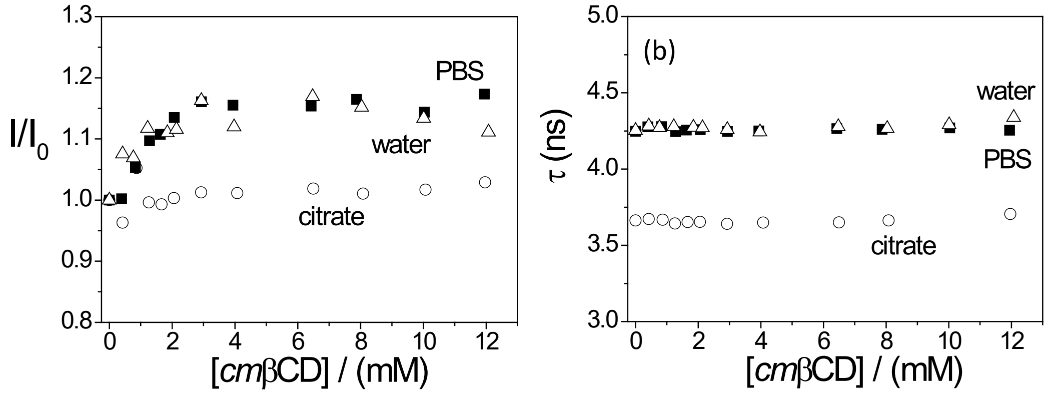


Figure S2. (a) Normalized fluorescence (*I/I*$_0$) where I and $I_0$ are the areas under emission spectra measured at each [*cm*βCD] for CPT/*cm*βCD solutions and CPT in the absence of *cm*βCD respectively. (b) Lifetime ($\tau$) of CPT/*cm*βCD solutions in absence and in presence of *cm*βCD at different concentrations in citrate buffer (○), PBS buffer (□) and water (△). T=25 ± 0.1 ºC.

**Section S3.** Influence of the environment on the quantum yield and fluorescence lifetime of CPT.

To investigate potential changes in fluorescence intensity and lifetimes of CPT upon complexation, fluorescence quantum yields ($\phi$) and lifetimes ($\tau$) were measured for diluted CPT solutions in media of varying polarity and viscosity. For this purpose, hydroxylated solvents and some of their mixtures with water were used (Figure S3). The inclusion of a guest inside a CD cavity is expected to affect both $\phi$ and $\tau$, as the guest is transferred from water, a highly polar medium with a dielectric constant ($\varepsilon$) close to 80, to the less polar inner cavity of βCD, whose $\varepsilon$ has been estimated to be around 50 [47]. Additionally, the microviscosity surrounding the complexed guest increases compared to the free guest. Changes in these parameters upon complexation are often exploited to easily obtain complexation constants [47,67,75-78]. However, $\phi$ and $\tau$ values measured for the dilute solutions of CPT in hydroxylated solvents revealed no clear correlation with $\varepsilon$ and $\eta$ parameters of the solvent (see Figure S3). Fluorescence quantum yields ($\phi$) for CPT solutions ranged from 0.63 to 0.68, slightly lower than the 0.72 reported by Thakur et al. in a CPT/PBS solution [79]. The $\tau$ values were around ~3.7 ± 0.3 ns across different solvents, with the carboxylate and lactone forms showing values of 4.5 ns and 3.7 ns, respectively. Only a minor decrease in $\tau$ was observed in less polar solvents, with little dependence on $\eta$ [42]. Overall, these findings demonstrate that the emission intensity and lifetime of CPT are relatively insensitive to the surrounding medium, as illustrated in Figure 1 of main text.

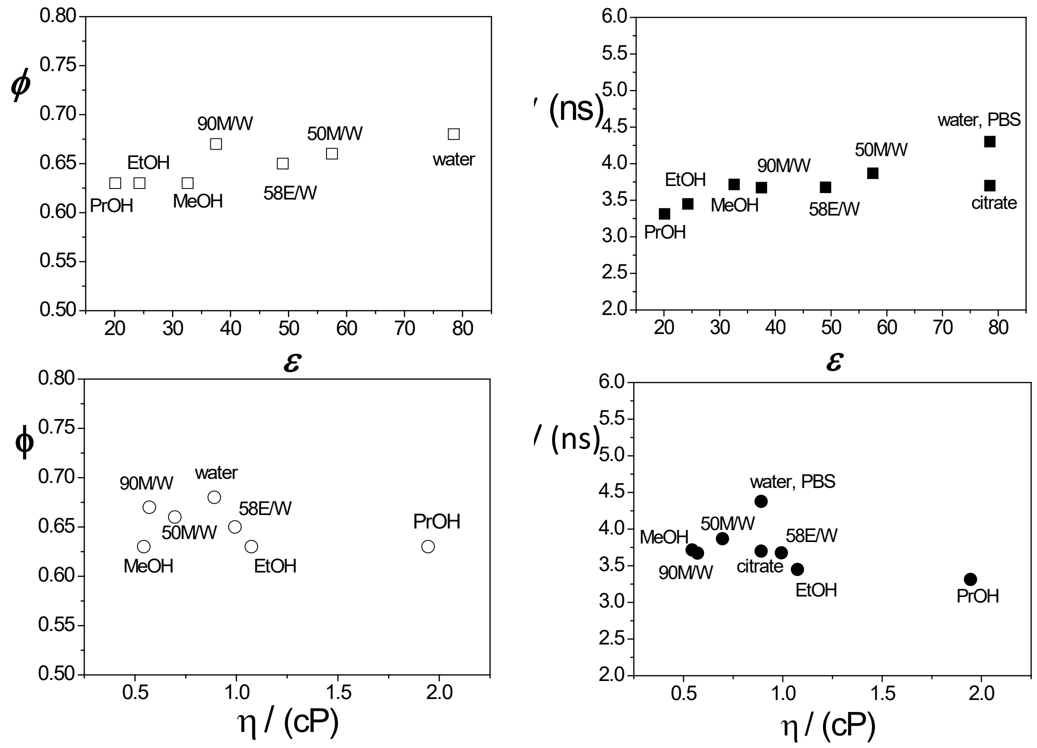


Figure S3. Fluorescence quantum yields (open symbols) and fluorescence lifetimes (full symbols) of CPT dilute solutions in methanol (MeOH), ethanol (EtOH), propanol (PrOH) and solvent mixtures: MeOH/$H_2O$ (50%), MeOH/$H_2O$ (90 %) and EtOH/$H_2O$ (58 %) (v/v) versus polarity (ε) (squares) and viscosity (η) (circles) of the solvents at 25 ± 0.1 ºC.

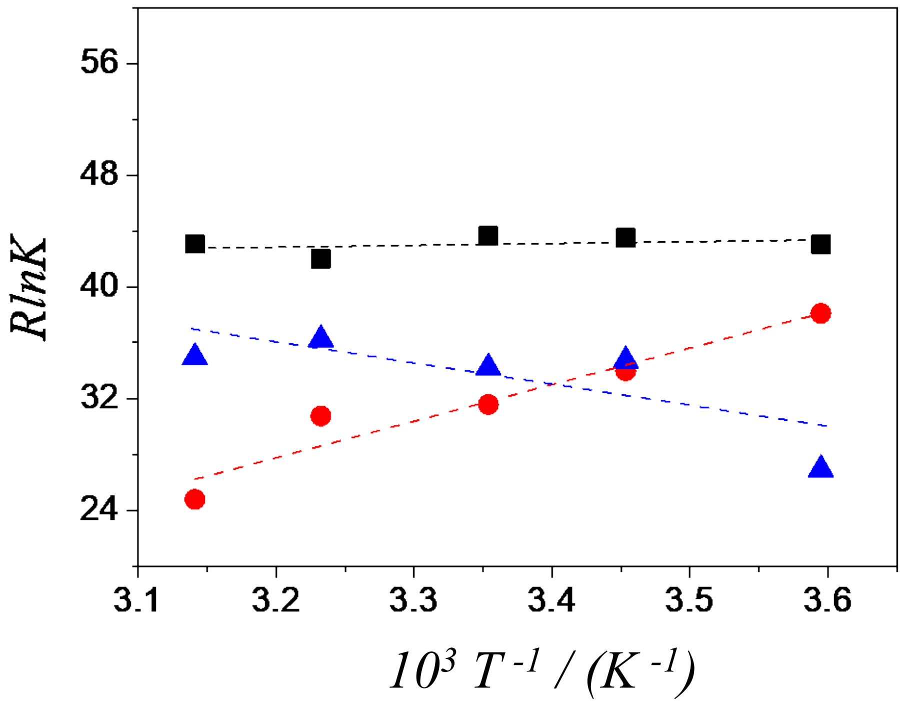


Figure S4. Van't Hoff representations using association constants values of Table 1 for the complexation of CPT with *cm*βCD in citrate buffer at pH=3.5 (black squares), PBS and water at pH=7.4 (red circles and blue triangles respectively)

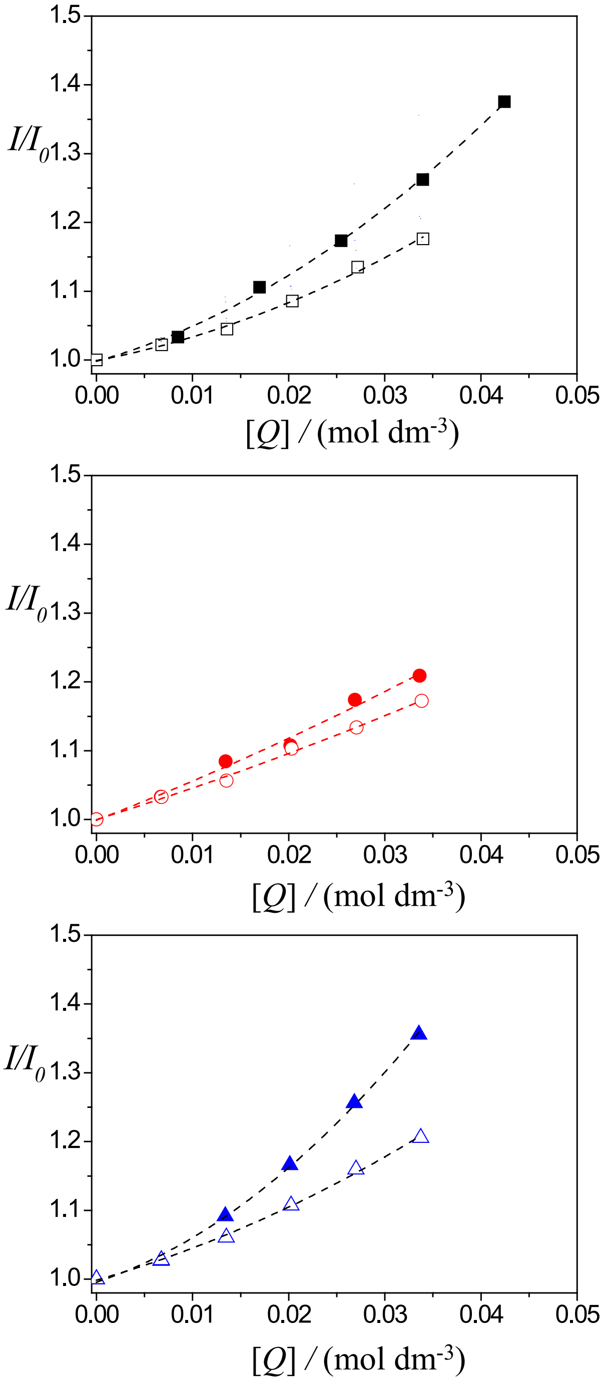


Figure S5. Stern-Volmer plots from fluorescence intensities for isolated CPT (filled symbols) and at the maximum concentration used for *cm*βCD (~12 $\times 10^{-3}$ $moldm^{-3}$) (open symbols) in citrate buffer (upper panel), PBS buffer (middle panel) and water (bottom panel). Quencher Q was diacetyl (2,3-butanedione). Measurements were performed at 25 ± 0.1 ºC.

**Table S1.** Stern–Volmer constants ($K_{SV}$), fluorescence lifetimes in the absence of diacetyl ($\tau_0$) and bimolecular quenching constants ($k_q$) for CPT in the absence and in the presence of *cm*βCD or *am*βCD in different solvents.

| Solvent | System | $K_{SV}$ / dm$^3$ mol$^{-1}$ | $\tau_0$ / ns | $kq \times 10^{-9}$ / dm$^3$ mol$^{-1}$ s$^{-1}$ |
|---|---|---|---|---|
| citrate (pH=3.5) | CPT | 6.9 | | 1.9 |
| | CPT + *cm*βCD | 4.1 | 3.7 | 1.1 |
| PBS (pH=7.4) | CPT | 6.4 | | 1.5 |
| | CPT + *cm*βCD | 5.1 | 4.3 | 1.2 |
| $H_2O$ (pH=7.4) | CPT | 8.4 | | 2.0 |
| | CPT + *cm*βCD | 5.3 | 4.3 | 1.2 |

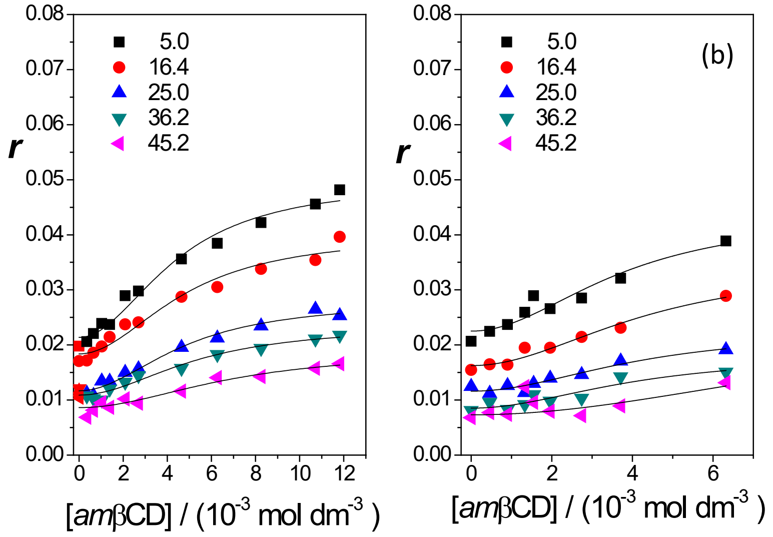


Figure S6. Fluorescence anisotropies, *r*, for CPT/*am*βCD solutions in PBS buffer (a) and water at pH=7.4 (b) at near 5 (black), 15 (red), 25, (blue), 35 (green) and 45 (magenta) ºC. The concentration range were 0-12 $\times 10^{-3}$ mol dm$^{-3}$ in PBS and 0- 6 $\times 10^{-3}$ moldm$^{-3}$ in water.

**Table S2.** Association constants ($K$) and $r_\infty$ values of the CPT and *am*βCD solutions in different media and temperatures obtained by fitting experimental values to Eq. S4 with n=2.

| PBS (pH 7.4) | | | $H_2O$ (pH 7.5) | | |
|---|---|---|---|---|---|
| **T /ºC** | $r_\infty$ | $K \times 10^3$ / $dm^6\,mol^{-2}$ | **T /ºC** | $r_\infty$ | $K \times 10^3$ / $dm^6\,mol^{-2}$ |
| 6.6 | 0.050 | 51.0 ± 12.1 | 5.0 | 0.046 | 54.7 ± 19.5 |
| 16.0 | 0.040 | 43.8 ± 14.3 | 15.0 | 0.035 | 49.9 ± 11.5 |
| 25.0 | 0.028 | 43.9 ± 10.2 | 25.3 | 0.023 | 48.8 ± 17.2 |
| 35.4 | 0.023 | 35.85± 11.6 | 35.4 | 0.018 | 47.9 ± 38.5 |
| 44.5 | 0.019 | 21.2 ± 12.8 | 44.4 | 0.028 | 8.8 ± 29.8 |

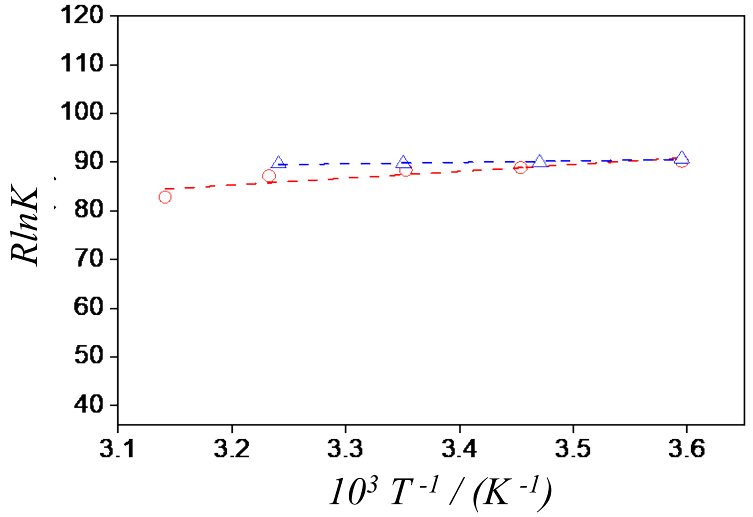


Figures S7. Vant'Hoff plots of Rln K vs $T^{-1}$ for the formation of CPT:*am*βCD complexes in PBS buffer (red circles) and water (blue triangles).

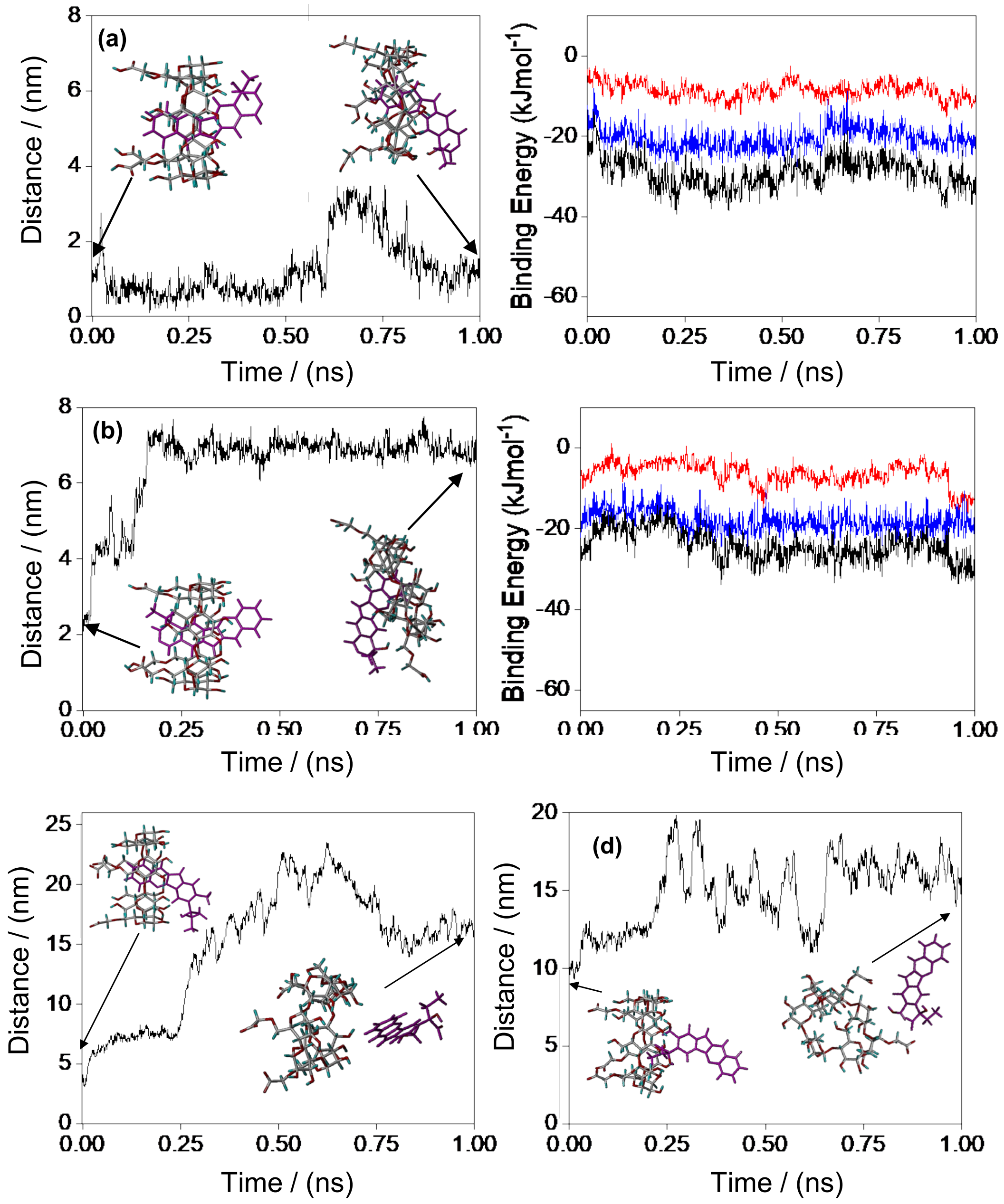


Figure S8. (a) Histories of the *oo′* distances between the center of mass of glycosidic oxygen atoms of *cm*βCD and the CPT (Figure S21) and binding energies (total binding in black, electrostatics in red and van der Waals in blue contributions), obtained from the analysis of MD simulations starting with the MBE structures of the CPT:*cm*βCD complexes obtained in MM (Figure 4 of the main text). Remember: for the approach of CPT to the secondary face of the uncharged *cm*βCD, either via the pyrrolo[3,4-β]-quinoline moiety (a) or the alpha-hydroxy lactone ring (b); idem for the approach of CPT to the secondary face of the charged (–3 esu) *cm*βCD, either via the pyrrolo[3,4-β]-quinoline moiety (c) or the open ring from the hydrolyzed lactone (carboxylate form) (d).

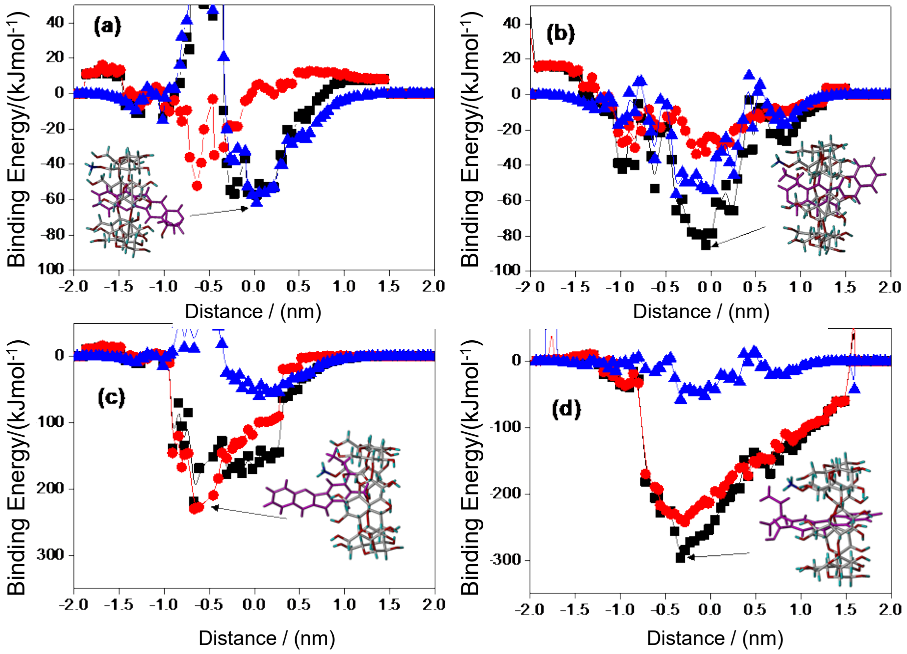


Figure S9. Variation of the total CPT–*am*βCD interaction energies (■), electrostatic (●) and van der Waals (▲) contributions as uncharged CPT approaches the secondary face of charged *am*βCD (+1 esu), either from the pyrrolo[3,4-β]-quinoline moiety (a) or the α-hydroxy lactone ring sides of CPT (b) to mimic the complexation at pH 3.5. Idem for charged (−1 esu) CPT approaching to the secondary face of charged *am*βCD (+1 esu), from the pyrrolo[3,4-β]-quinoline (c) or the carboxylate (d) sides of charged CPT (−1 esu) mimicking the complexation at pH 7.4. Inset are the minima binding energy (MBE) structures indicated by the arrows.

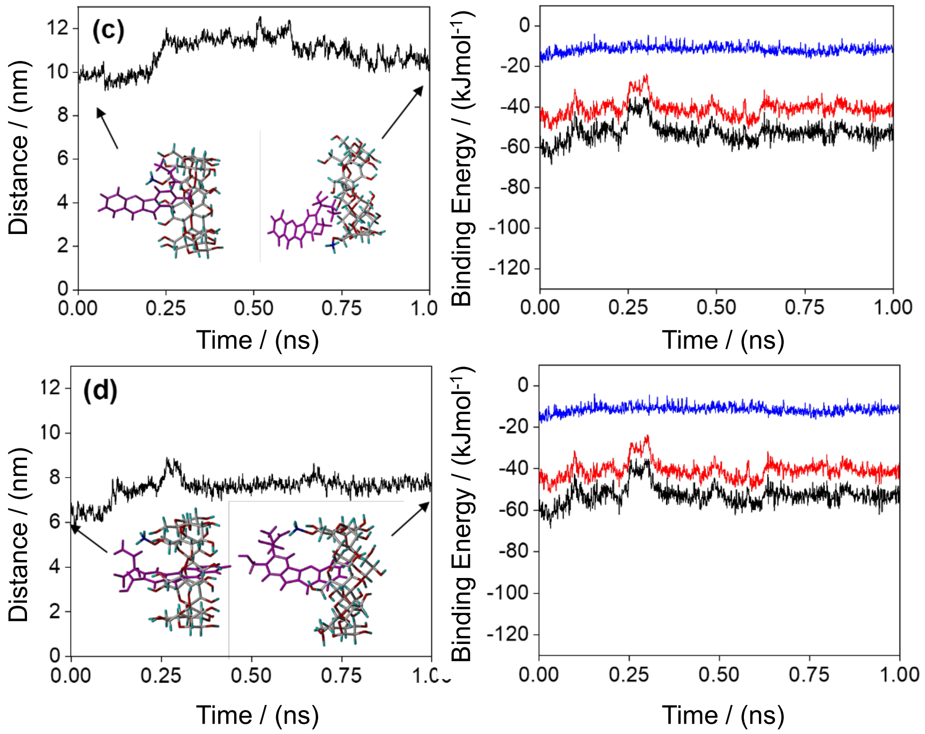


Figure S10. Histories of the *oo´* distances between the center of mass of glycosidic oxygen atoms of *am*βCD and the CPT (Figure S22) and binding energies (total binding in black, electrostatics in red and van der Waals in blue contributions), obtained from the analysis of MD simulations starting with the MBE structures of the CPT:*am*βCD complexes obtained in MM (Figure S9c and d) mimicking complexation at pH 7.4; Remember: for the approach of CPT to the secondary face of the protonated *am*βCD, either via the pyrrolo[3,4-β]-quinoline moiety (c) or the open ring from the hydrolyzed lactone (carboxylate form) (d).

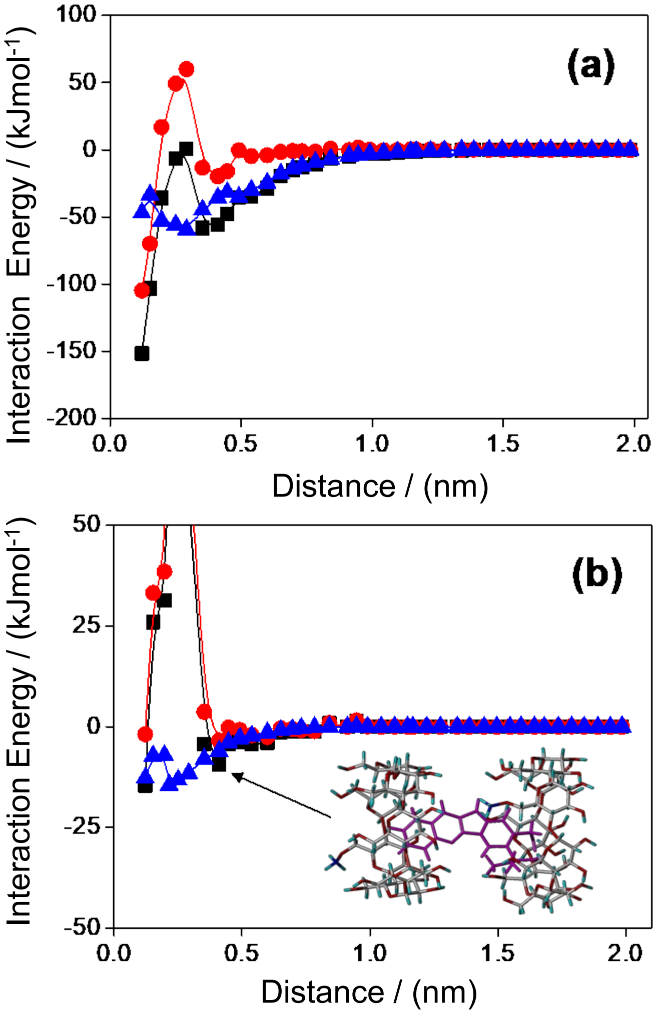


Figure S11. (a) Variation of the total (CPT:*am*βCD)–*am*βCD interaction energies (■), along with electrostatic (●) and van der Waals (▲) contributions, as *am*βCD approaches the preformed CPT:*am*βCD 1:1 complex of charged *am*βCD (+1 esu) shown in Figure S10c following the approach scheme depicted in Figure S23. (b) Variation of the total *am*βCD–*am*βCD interaction energies (■), along with electrostatic (●) and van der Waals (▲) contributions during the formation of the 1:2 complex.

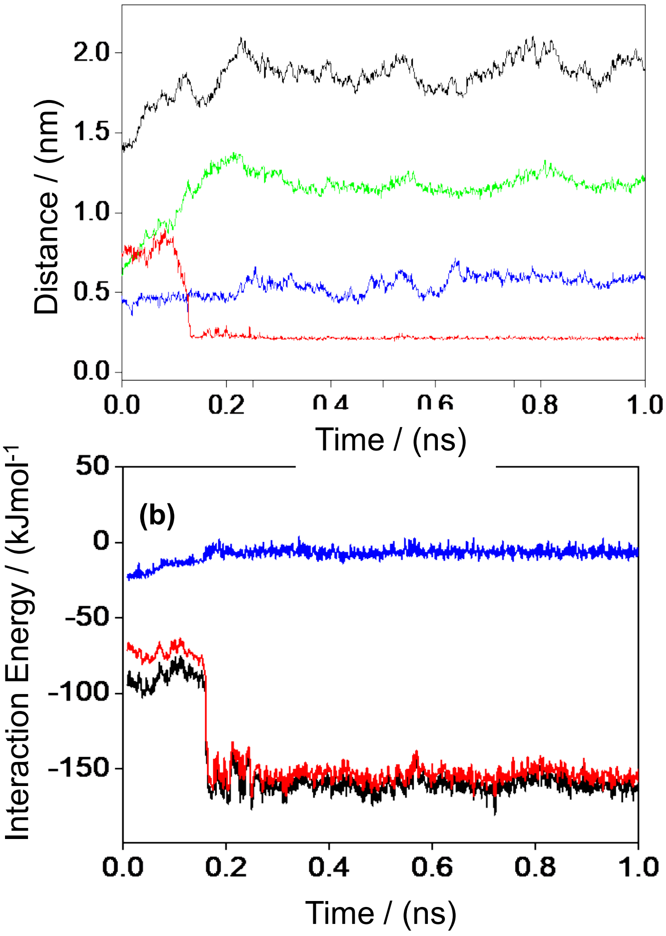


Figure S12. Histories of several distances: (a) between center of mass of glycosidic oxygen atoms of both *am*βCDs (black); between nitrogen –$(NH_3)^+$ from nearest *am*βCD and carbon atom of the carbonyl group of the carboxylate from CPT (red) and the *oo'* CPT to each *am*βCD1 (blue) or *am*βCD2 (green) distances; (b) Histories for the CPT– (*am*βCD)$_2$ interaction energies (total in black, electrostatics in red and van der Waals in blue contributions), obtained from the analysis of MD simulations starting with the MBE structure of the CPT:(*am*βCD)$_2$ complex depicted inset in Figure S11b.

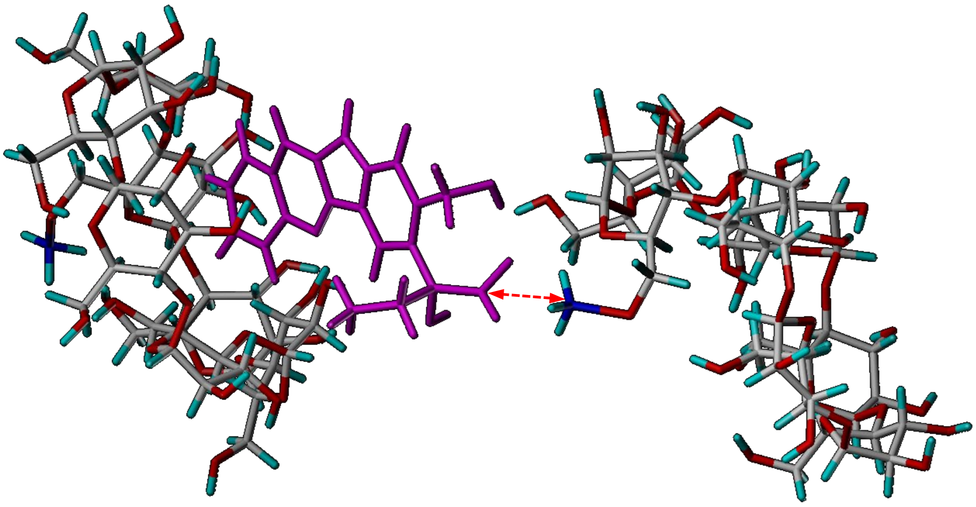

Figure S13. Structure for the CPT:($am$βCD)$_2$ complex at the end of the molecular dynamic trajectory. By the arrow is shown the distance between nitrogen $-(NH_3)^+$ from nearest $am$βCD and carbon atom of the carbonyl group of the carboxylate moiety from CPT.

**Section S14.** Protocols for CPT solubility studies as a function of *cm*βCD concentration. To conduct the solubility study of CPT as a function of *cm*βCD concentration, 3 mL of *cm*βCD solutions at concentrations of 2, 5, 8, 10, 14, and 16 $\times 10^{-3}$ moldm$^{-3}$ were prepared. To each *cm*βCD solution, a solution of CPT (5 mg) in methanol (0.2 mL) was added to achieve saturation. The samples were then filtered using a 0.45 μm nylon filter to remove undissolved drug. For reference, a saturated solution of CPT was prepared without *cm*βCD. The concentration of CPT in the filtered solutions was determined by measuring the absorbance (at 358 nm or 370 nm at pH 3.5 or 7.4 respectively).

**Section S15.** Phase-solubility diagrams protocols. To obtain solubility phase diagrams, *cm*βCD solutions were prepared at concentrations ranging from 2 to 16 $\times 10^{-3}$ moldm$^{-3}$ at two different pH values (3.5 and 7.4), while maintaining a constant drug concentration using saturated solutions. After preparation, the solutions were stirred overnight, filtered, and the concentration of the solubilized drug was calculated based on the absorption maxima. For a drug:host 1:1 complex with good solubility, CPT concentration as a function of the [*cm*βCD] representations are linear and the association constants can be obtained from the slope, *m*, and the intercept, $S_0$ by the equation:

$$K_{drug:host} = m \big/ \left[ S_0 \left( 1 - m \right) \right] \quad \text{(S6)}$$

where $S_0$ is the solubility of the drug [48].

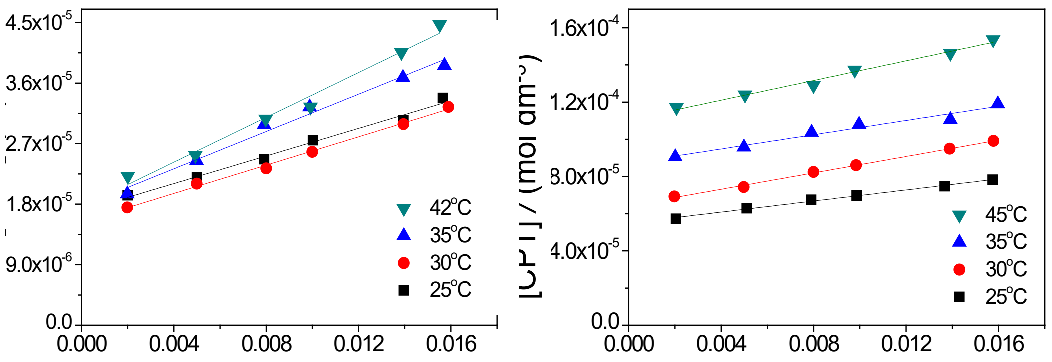


Figure S15. Solubility diagrams of CPT in the presence of *cm*βCD at different temperatures and two pH values.

Table S3. Association constants ($K$) from solubility phase diagrams of Figure S15. Solubility of free CPT drug ($S_0$) at each pH and temperature were obtained in other experiments (standard deviation in $K$ values of ~10%).

**pH=3.5**

| T /ºC | *(m, slope)* | $R^2$ | $S_0$ /moldm$^{-3}$ | $K$ /mol$^{-1}$dm$^3$ |
|---|---|---|---|---|
| 25 | 0.0010 | 0.99 | $4.82 \cdot 10^{-6}$ | 208 |
| 30 | 0.0011 | 0.99 | $5.17 \cdot 10^{-6}$ | 213 |
| 35 | 0.0014 | 0.98 | $5.88 \cdot 10^{-6}$ | 238 |
| 42 | 0.0017 | 0.98 | $9.26 \cdot 10^{-6}$ | 184 |

**pH=7.4**

| T /ºC | *(m, slope)* | $R^2$ | $S_0$ /moldm$^{-3}$ | $K$ /mol$^{-1}$dm$^3$ |
|---|---|---|---|---|
| 25 | 0.0015 | 0.99 | $2.76 \cdot 10^{-5}$ | 54 |
| 30 | 0.0022 | 0.99 | $5.13 \cdot 10^{-5}$ | 43 |
| 35 | 0.0019 | 0.96 | $6.28 \cdot 10^{-5}$ | 32 |
| 42 | 0.0026 | 0.98 | $8.86 \cdot 10^{-5}$ | 29 |

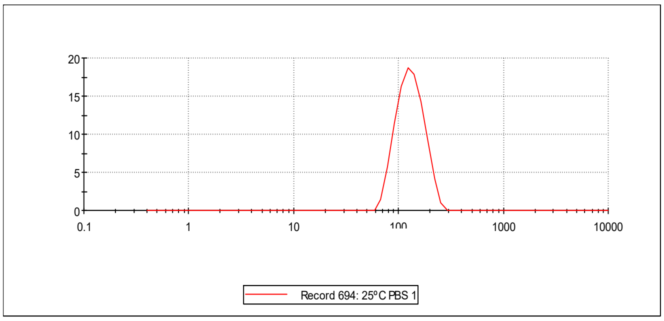


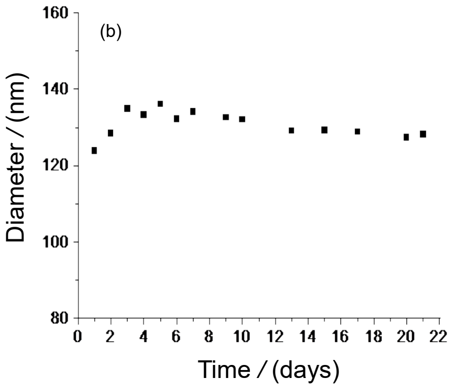


Figure S16. (a) Dynamic light scattering (DLS) measurements of liposomes dispersed in PBS buffer at 25 ± 0.1°C. Three independent measurements were conducted over a 21-day period to monitor the evolution of the hydrodynamic diameter. The plot shown corresponds to one representative measurement of freshly prepared liposomes, illustrating the intensity-weighted hydrodynamic diameter distribution at 25 °C. The mean diameter was 133±40 nm (intensity-weighted average).(b) Time-dependent variation of the mean hydrodynamic diameter of liposomes stored in PBS at 25 °C over 21 days.

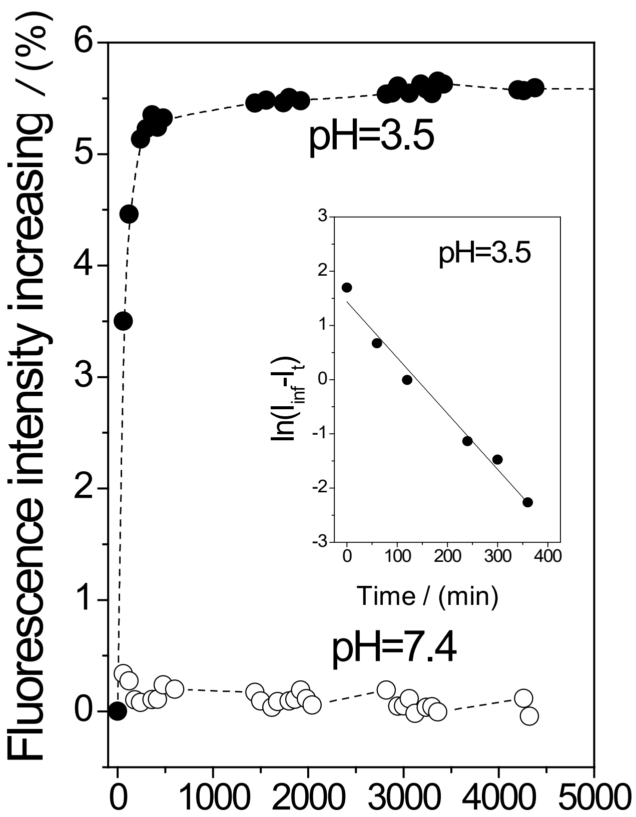


Figure S17. Time-dependent fluorescence intensity increase (%) representing CPT release from liposomes at pH 3.5 (●) and pH 7.4 (○). The system exhibits pH-responsive behavior with significant release at acidic conditions. Inset: Linearized first-order kinetic plot for pH 3.5, showing $\ln(I_{inf} - I_t)$ vs. time (min). The linear correlation confirms that the release of CPT follows first-order kinetics ($k=0.0103$ $min^{-1}$, $\tau_{1/2}=67.3$ min)

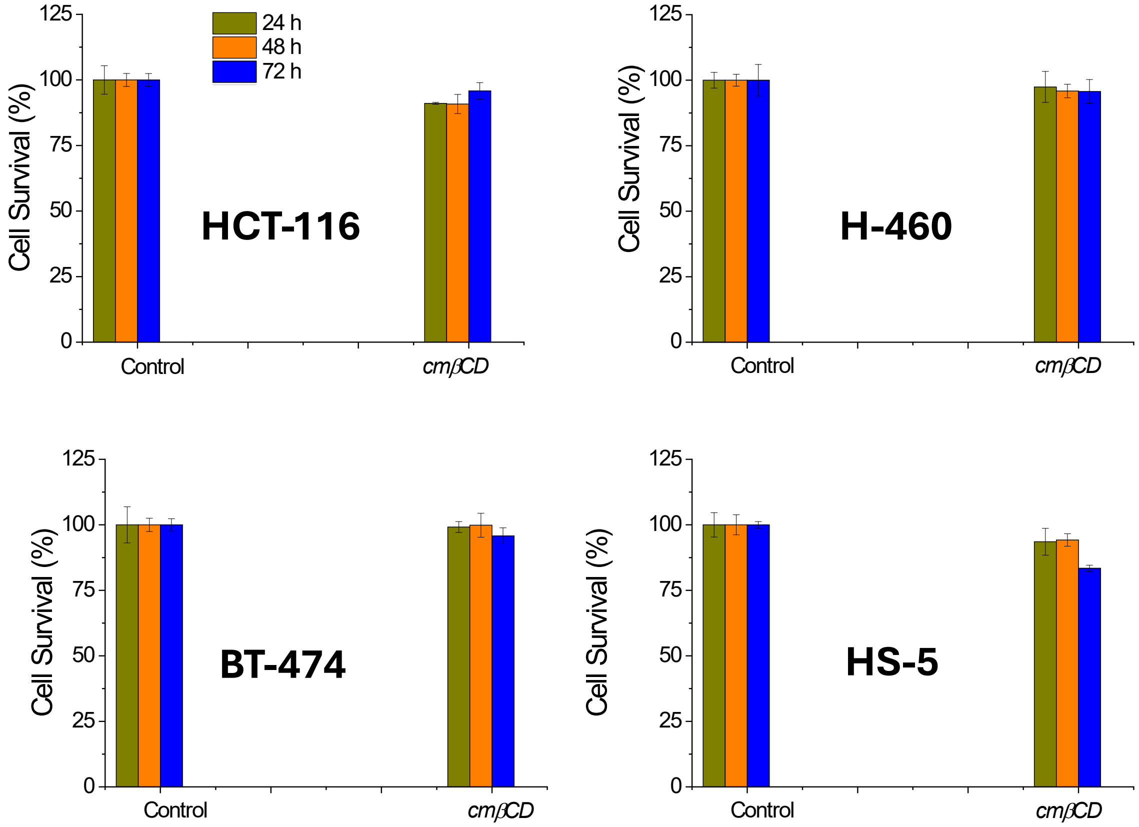


Figure S18. Cellular viability after exposing the different cell lines to *cm*βCD at a concentration of 1 mg/mL (0.65 $\times 10^{-3}$ moldm$^{-3}$).

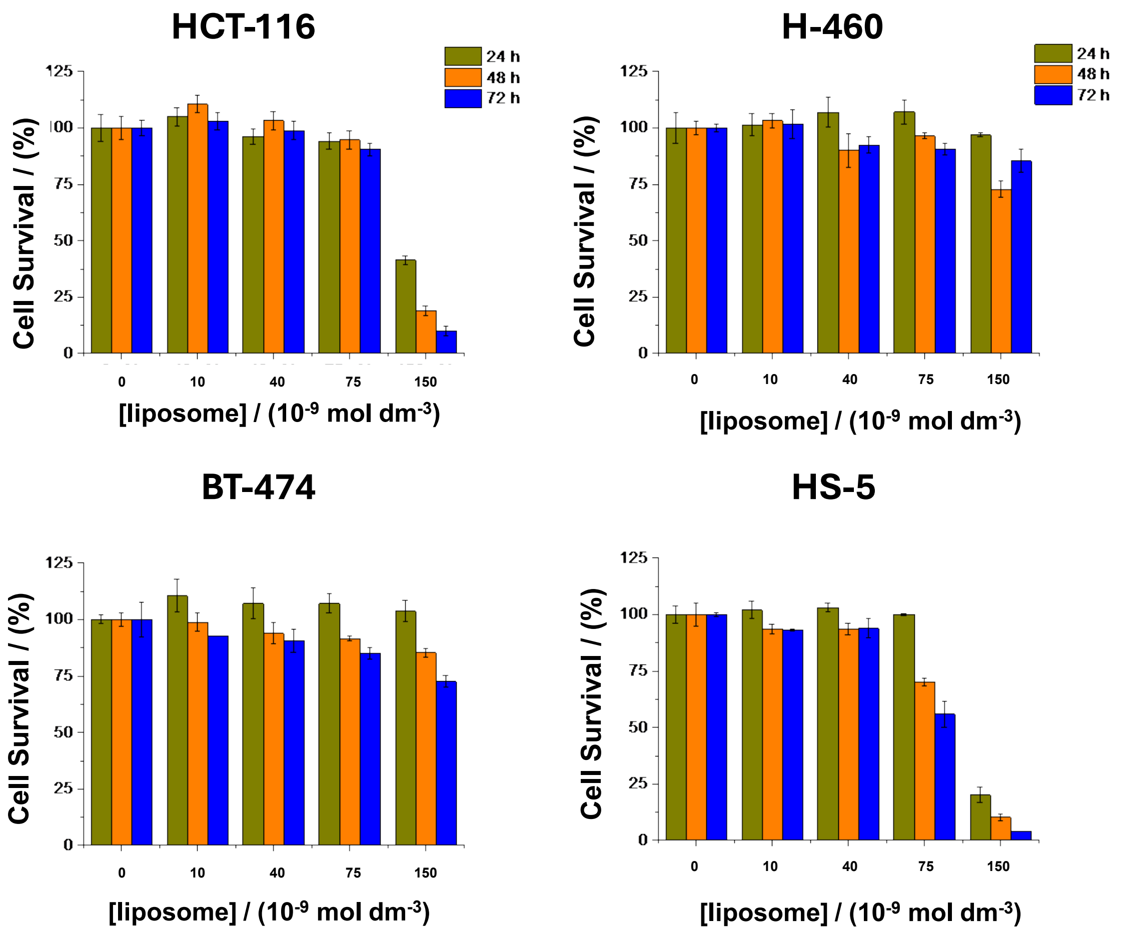


Figure S19. Cellular viability after exposing the different cell lines to different liposome concentration necessary to attain the CPT concentration of X scale.

**Section S20.** Protocols for Cell Lysosome Staining. Preparation of Culture Medium with LysoTracker Red: To visualize cellular lysosomes and evaluate the internalization of liposomes, lysosomes were stained using LysoTracker Red. A $100\times10^{-6}$ $moldm^{-3}$ stock solution of LysoTracker Red was prepared in PBS (pH 7.4). From this stock, a working solution was made by diluting the dye to a final concentration of $100\times10^{-9}$ $moldm^{-3}$ in DMEM culture medium. For staining, 1 mL of the working solution was added to each cell culture plate.

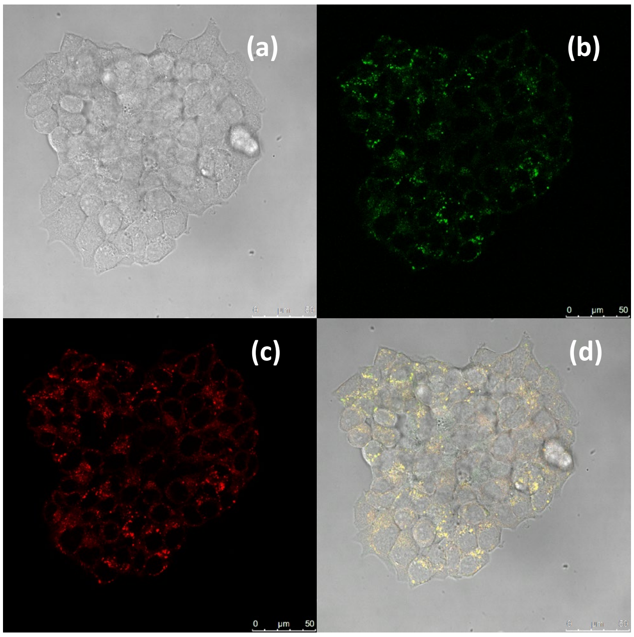


Figure S20. Fluorescence confocal images: a) Bright field image. b) Fluorescence image (excitation at 488 nm). c) Fluorescence image (excitation at 570 nm). d) Overlay images a, b and c. Liposomes concentration 115 mg/mL.

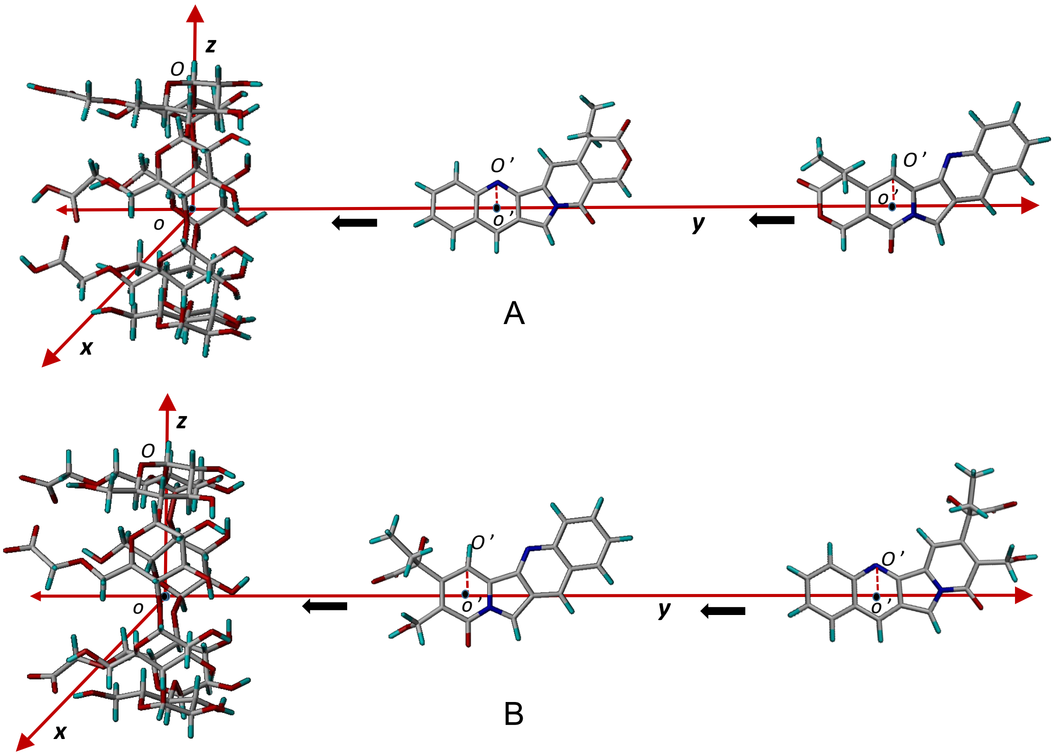


Figure S21. (A) Scheme for the approach of CPT to the secondary face of the uncharged *cm*βCD, either via the pyrrolo[3,4-β]-quinoline moiety or the alpha-hydroxy lactone ring, simulating conditions at pH 3.5 (net system charge of 0 esu). (B) Similarly, for the approach of CPT to the secondary face of the charged (–3 esu) *cm*βCD, either via the pyrrolo[3,4-β]-quinoline moiety or the open ring from the hydrolyzed lactone (–1 esu), simulating conditions at pH 7.4 (net system charge of –4 esu).

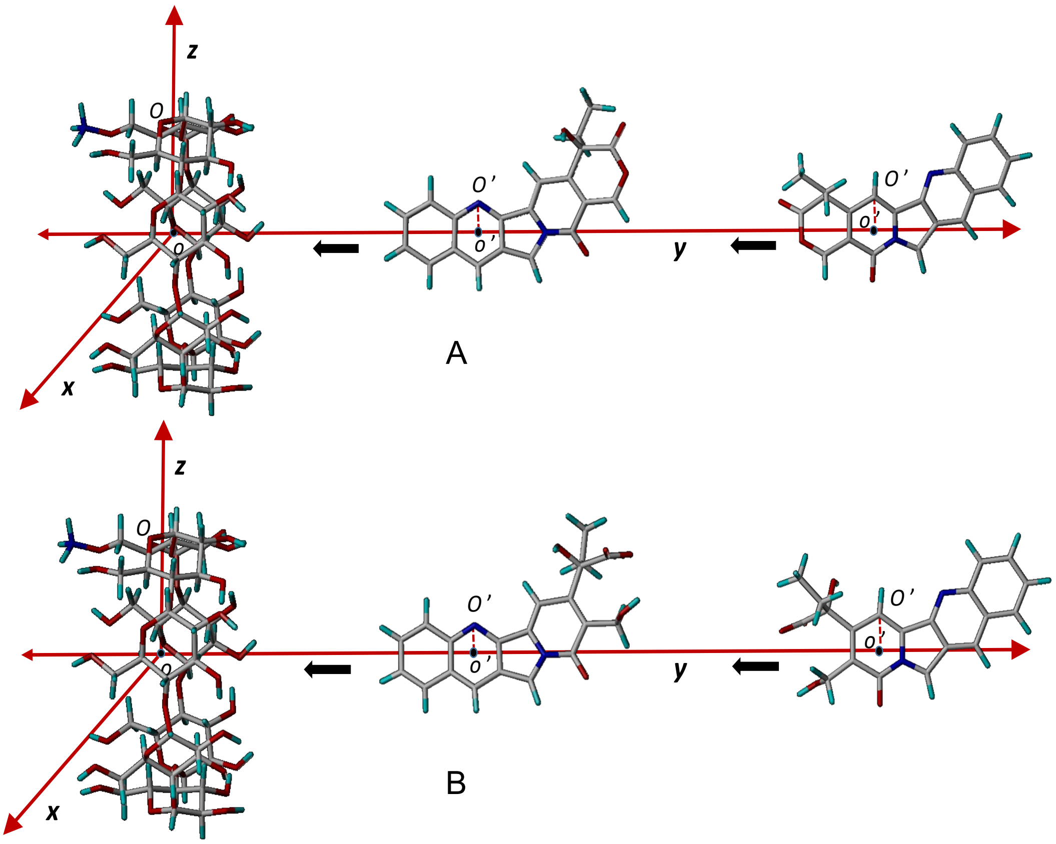


Figure S22. (A) Scheme for the approach of CPT to the secondary face of the charged (+1 esu) *am*βCD, either via the pyrrolo[3,4-β]-quinoline moiety or the alpha-hydroxy lactone ring, simulating the conditions at pH 3.5 (net system charge of +1 esu). (B) Similarly, for the approach of CPT to the secondary face of the charged (+1 esu) *am*βCD, either via the pyrrolo[3,4-β]-quinoline moiety or the open ring from the hydrolyzed lactone (−1 esu), simulating the conditions at pH 7.4 (net system charge of 0 esu).

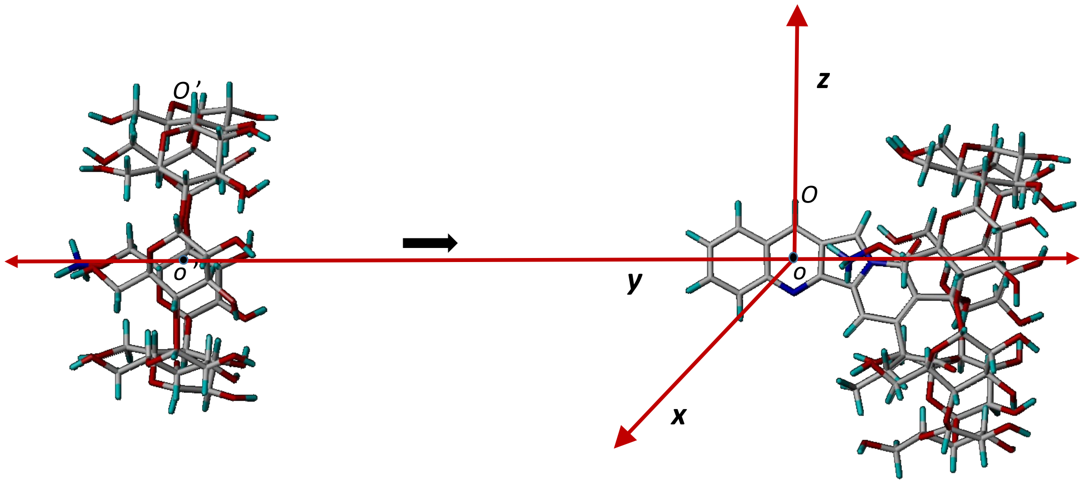


Figure S23. Scheme for the approach of a second amβCD molecule to the CPT βCD MBE structure of the (1:1) complex via the pyrrolo[3,4-β]-quinoline moiety, simulating the formation of a 1:2 stoichiometric complex at pH 7.4 (with a net system charge of +1 esu).
